# Occupational respiratory disorders in Iran: a review of prevalence and inducers

**DOI:** 10.3389/fmed.2024.1310040

**Published:** 2024-02-08

**Authors:** Sima Beigoli, Fatemeh Amin, Hamideh Kazemi Rad, Ramin Rezaee, Mohammad Hossein Boskabady

**Affiliations:** ^1^Applied Biomedical Research Center, Mashhad University of Medical Sciences, Mashhad, Iran; ^2^Department of Physiology, Faculty of Medicine, Mashhad University of Medical Sciences, Mashhad, Iran; ^3^Physiology-Pharmacology Research Center, Research Institute of Basic Medical Sciences, Rafsanjan University of Medical Sciences, Rafsanjan, Iran; ^4^Department of Physiology and Pharmacology, School of Medicine, Rafsanjan University of Medical Sciences, Rafsanjan, Iran; ^5^International UNESCO Center for Health-Related Basic Sciences and Human Nutrition, Faculty of Medicine, Mashhad University of Medical Sciences, Mashhad, Iran

**Keywords:** occupational, noxious agents, workplace environmental pollution, exposure, respiratory disorders, Iran

## Abstract

The link between occupational respiratory diseases (ORD) and exposure to harmful factors that are present in the workplace has been well shown. Factors such as physical activity, age and duration of occupational exposure playing important roles in ORD severity, should be identified in the workplace, their effects on workers health should be studied, and ultimately, exposure to them must be minimized. We carried out a literature review by searching PubMed, Scopus, and Web of Science databases to retrieve studies published from 1999 until the end of April 2023 reporting the prevalence and inducers of ORD in Iran. In Iranian workers, several ORD such as interstitial lung disease, silicosis, occupational asthma, pulmonary inflammatory diseases, chronic obstructive pulmonary diseases, and lung cancers have been reported. It was indicated that ORD mainly occur due to repeated and prolonged exposure to noxious agents in the workplace. We also extracted the prevalence of ORD in different regions of Iran from the retrieved reports. Based on our literature review, the prevalence of ORD among Iranian workers highlights the importance of regular assessment of the risk of exposure to noxious agents in the workplace to develop measures for preventing potential adverse effects.

## Introduction

Through epidemiological surveys, infectious and some chronic diseases can be effectively controlled and prevented. However, monitoring programs are hardly ever implemented for controlling and preventing work-related diseases despite the high social and economic impact of occupational diseases (1). Prolonged exposure to certain toxic agents entering the lungs causes work-related lung diseases. Even after termination of exposure, these lung diseases may continue. Exposure to toxic compounds in the workplace may cause lung diseases such as chronic obstructive pulmonary disease (COPD), lung cancer, asthma, and pleural disease (2). The global prevalence, of ppneumoconiosis and other chronic occupational respiratory diseases (ORD) is 453,000 and 2,631,000 cases/year, respectively (3). For example, 14,000 deaths from coal pneumoconiosis, 9,000 from silicosis, and 7,000 from asbestosis were reported globally in 2000 (3).

Mineral fibers of asbestos include silicates bound to other elements, predominantly magnesium and iron. Across the country, asbestos is still found in homes, buildings, roads, factories, schools, ships, automobiles and trains. Asbestos was being combined with thousands of constructions as well as household and commercial products, all over the 20th century. Fireproof coatings, cement, and concrete, bricks, gaskets, pipes, insulation, flooring, drywall, roofing; joint compound, sealants, and paints are among these products. The main cause of global work-related deaths is asbestos exposure which was confirmed to cause cancer as well as other serious diseases such as mesothelioma, lung cancer, and asbestosis (4). Asbestos-based occupational disease is still a main occupational health issue, particularly in developing countries which lack effective control of exposure (5).

Allergic agents are among the most important inducers of ORD (6). Thus, the existence of these agents in the workplace needs to be recognized as early as possible. Moreover, the ORD risk caused by these agents needs to be evaluated to take necessary actions. Certain occupational factors may cause occupational diseases depending on metabolism and sensibility and delayed responses may be induced following occupational exposure (6). Therefore, if a worker presents with unknown respiratory conditions, immediate medical examination by a physician should be done and occupational exposure should be considered.

In the workplace, the airways such as nares and alveoli have contact with 14,000 liters of air for 40 h/week work (7). More importantly, ORD is considerably affected by pollution severity, physical activity, and exposure period length (8). Thus, workers exposed to occupational pollutants for longer period of time, are at a higher ORD risk (9). Ventilation is increased by physical activity and the breathing is shifted from nasal to oral-nasal, thus, air particle filtration through the airways is reduced and the lower airways are more exposed to airborne noxious agents (10). Although strong irritants can induce severe responses in the workplace, less sensitizing agents might be inhaled for longer periods and a lung injury is produced. Thus, occupational exposure to noxious agents is a key contributor to respiratory illnesses (11).

Amongst ORD, occupational sensitizer-induced asthma happens after exposure to excessive quantities of irritant particles in the workplace (12). Among the inducers of allergic occupational asthma are low molecular weight (LMW) antigens such as chemicals and metals, and high molecular weight (HMW) antigens such as glycopeptides, and proteins produced by plants, animals, and microbes (12). The most common causes of conventional occupational asthma are HMW antigens, however, LMW antigens should be also considered (13).

New materials are introduced to the workplace every year (14) and ORD is also occasionally caused by working in hospitals and office buildings (15). It was indicated that bakers, kitchen workers, waste collectors, cleaners, healthcare staff, hairdressers, farmers and textile industry workers suffer from occupational allergic disorders (16). Among the individuals working in the cement factory, pulmonary function tests (PFT) results such as forced expiratory volume in one second percent (FEV1%), forced vital capacity percent (FVC%) could be reduced by exposure to crystalline silica, resulting in restrictive pulmonary disorders (17). Moreover, reduction of PFT values were found among hairdressers (18), printing and bakery workers, and miners even in the first five years of employment (19).

**Figure 1:**
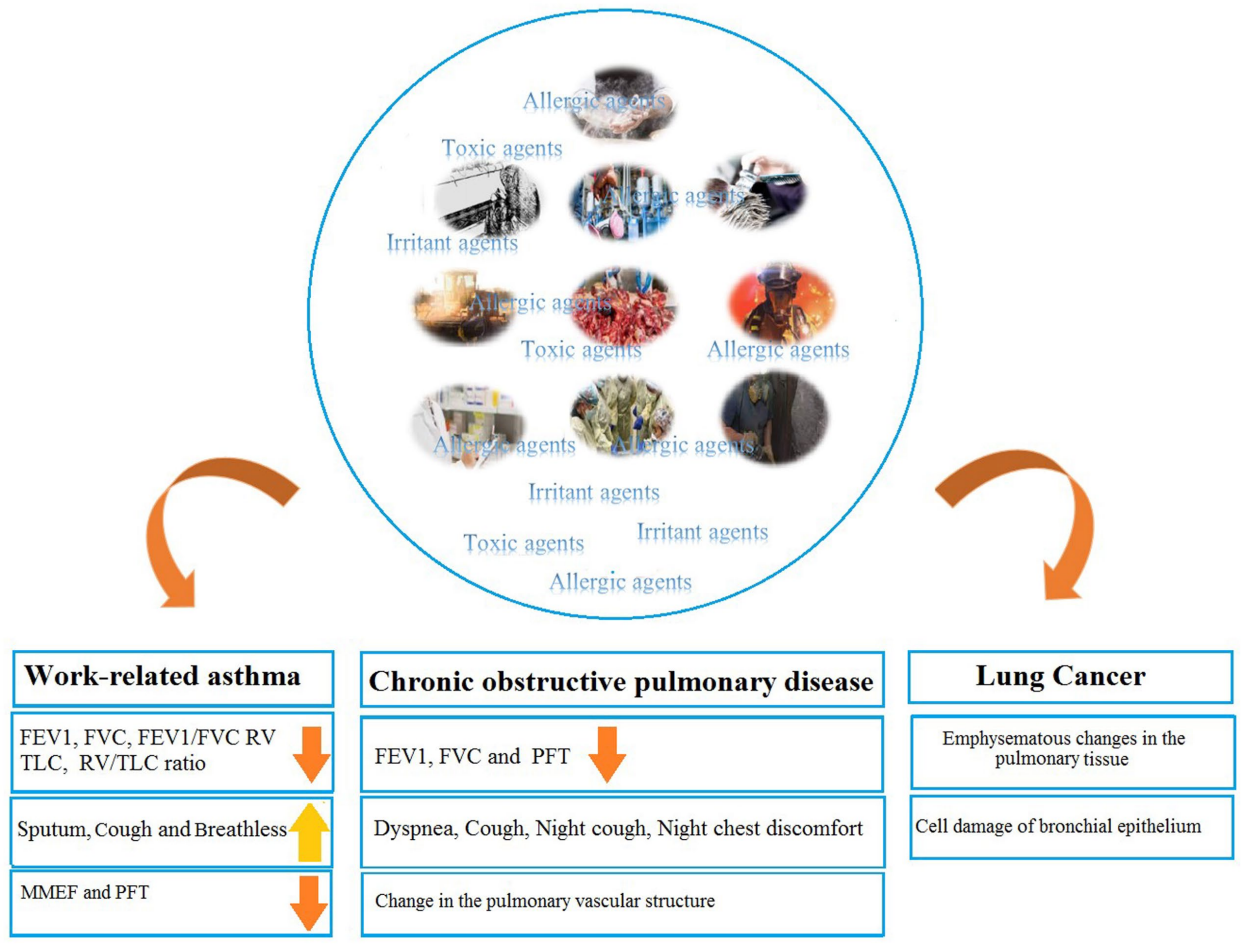
Occupational respiratory disorders.

In the present article, we reviewed ORD and ORD inducers in Iranian workers practicing various occupations (Figure 1).

## Methods

To prepare the present review, Scopus, PubMed, and Web of Science databases were searched through the keywords “job,” “occupation,” “toxic agents,” “allergic agents,” “environmental pollution,” “irritants agents,” “pulmonary function tests,” “respiratory symptoms,” “respiratory disorders,” “lung inflammation,” and “prevalence” for retrieving reports published from 1999 until the end of April 2023. In the present work, the papers on work-based respiratory diseases written in English were considered and the reference list of the collected articles was assessed.

**Figure 2:**
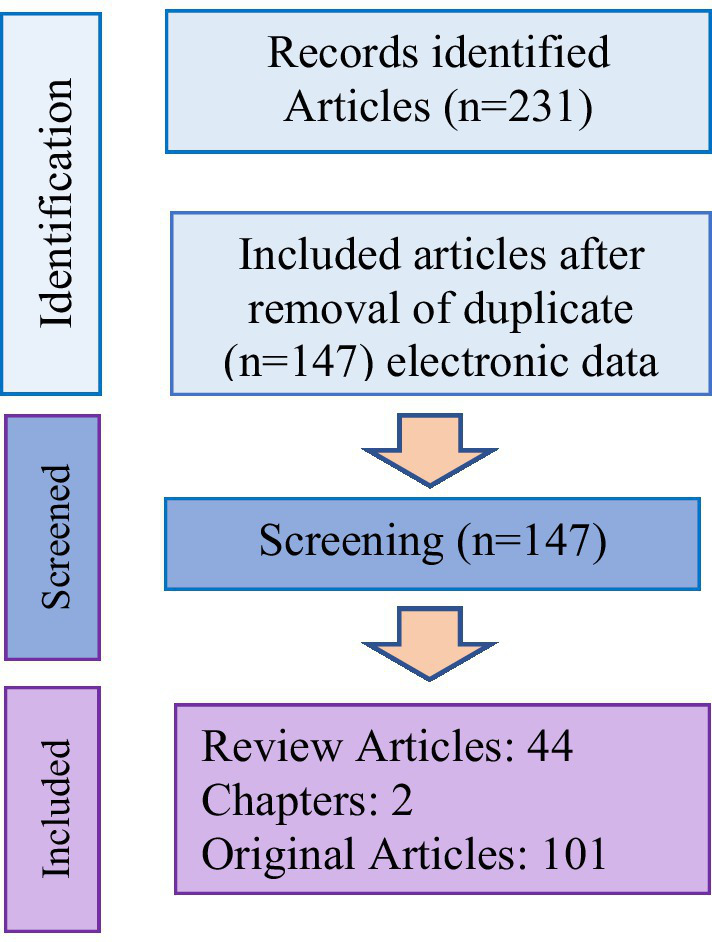
Flowchart of the process for selecting studies for the review.

Nevertheless, the studies with inadequate information, non-English and unpublished articles, abstracts, and letters to editors were removed. In total, among the initial 231 articles, 147 papers (44 review articles, 2 book chapters, and 101 original articles) were included in this review and 84 articles were duplicates (Figure 2).

## Results

### Prevalence of occupational respiratory disorders in Iran

Several respiratory diseases such as lung cancer, interstitial lung disease (ILD), silicosis, asbestosis, occupational asthma, inflammatory lung disease, COPD and lung cancer are induced by occupational factors (20). Exposure to pollutants in the work environment can exacerbate a person’s illness and having a history of occupational pollutants exposure is a crucial risk for work-related lung diseases. Emergence of new causes for occupational lung diseases in recent years reinforces the need to adhere to the principles of respiratory protection (20).

The prevalence of occupational COPD among the workers working in Kerman, Iran factories, exposing to dust, fog, and asbestos agents, was 27.3%, 13.3%, and 0.05%, respectively (20). In another study, using an occupational questionnaire, industrial workers in Tehran, Iran, were assessed for the effects of exposure to biomass fuels at various factories. The findings indicated a COPD prevalence of 9.2% among these workers (21).

Using a national recording system, in 2005, 5 cases of mesothelioma were reported due to asbest exposure (22). Occupations that are directly related to asbestos, such as asbestos factories, mineworkers, and many other professions, are exposed to high levels of this material in building and construction. Workers of shipyards, electrical industry, pulp and paper industry, sugar refineries, oil refineries, cigarette filter, and jewelry industry are also at risk, resulting in a higher prevalence of mesothelioma in these environments (23). In addition, a strong association between asbestos exposure and mesothelioma suggests the influence of asbestos exposure on mesothelioma incidence (22).

Kerman is one of the industrial provinces of Iran. Evidence shows that the prevalence of dust among 67 stone carvers in stone carvers of Kerman with inappropriate ventilation of working environment was about 26.9% (24). Significantly increased prevalence of respiratory symptoms among workers in industries of south Tehran, Iran, was shown and the prevalence of respiratory symptoms among exposed workers (dust, fume) was 30.9% (25).

A high prevalence of mesothelioma (17.1%) in various occupations such as oil company, construction, farmer, and carpet-weaver in southern Iran was reported. Table 1 shows the prevalence of ORD in Iran and Table 2 shows agents or worksite allergens, irritants, and toxic agents.

**Table 1:** The prevalence of occupational respiratory disorders in Iran.

Occupations	City	Study design	Exposed agents	Prevalence rate	Refs
Industries	Kerman	COPD	Dust	27.3%	(20)
		Asthma	Smoke & fog	13.3%	
Industries	Tehran	Mesothelioma	Biomass	9.2%	(21)
	Kerman		Dust	26.9%	(24)
	Tehran	Mesothelioma	Asbestos	10%	(23)
			Dust, fume, gas	22.8%	(25)
			Asbestos	31.7, and 30%	(22)
				0.05%	(20)
Construction	Southern Iran			31.4%	
Oil Company workers			Fuel-fired	3.3%	

COPD: Chronic obstructive pulmonary disease.

**Table 2:** Agents or worksites allergen, toxic, and irritant.

Occupation	Allergens and irritants	Toxins	Refs
Bakers	α-amylase, cellulose, hemicellulose, malt enzymes, egg powder, milk powder, sugar, Flour dust, wheat, biomass fuels	–	(26)
Hairdressers	Dyes, spray paint, persulphates	Sulfur dioxide	(27–29)
Agriculture	Plant products, flour dust, Animal products	Paraquat	(28–30)
Carpet weavers	Wood dust, Amines	–	(31)
Healthcare workers	Antimicrobials and biocides	Methacrylate, nickel and beryllium	(32–34)
Lifeguards	Chlorine	–	(35)
Factory workers	Cement dust, ceramic	Asbestos, crystalline silica, mustard gas, sulfur dioxide, cement dust, isocyanates, tile	(3, 4, 29)
Miners	Stone dust	Coal mines, lead, kaolin mine, gold mine, quarry, metallurgic industry	(29, 36)
Welding	–	Welding fumes	(37–39)

### Inducers of ORD in Iran and their mechanism of action

#### Allergic and irritant agents-induced ORD

Enzymes and wheat for bakeries workers, antimicrobials, latex, and biocides for healthcare workers, isocyanates, cobalt and nickel for metalworkers, and persulfates for hairdressers are some of the most prevalent allergens present in the work places. Normally, occupational allergens are categorized as LMW <5 kDa and HMW >5 kDa. It was found that the size of allergens and irritant materials has a key role in their allergen city and the underlying action mechanism (27).

The frequency of occupational rhinitis is three times higher than occupational asthma, which is often related to occupational conjunctivitis like itching, irritation tearing, and red eyes (27). Also, there is evidence on irrigation agents leading to occupational COPD or occupational asthma due to exposure to irritant agents in the work place and environment (40). A relationship was found between asthma symptoms in domestic cleaning and exposure to bleach as well as other possible irritant agents (41). Using irritant cleaning products may have a considerable impact on public health since these products are used commonly both at home and in the workplace. According to the community-based studies, cleaning workers are at higher risk of asthma as well as other respiratory symptoms; however, the responsible agents are not clear (41). The irritants have potentially higher health impacts since these agents can induce asthma and persistent symptoms and affect PFT values (42). Table 3 shows ORD induced by allergic and irritant agents in Iran.

**Table 3:** Occupational respiratory disorders (ORD) induced by allergic and irritant agents in the Iran.

Occupational	Study design	Agents	Effects	Refs
Agriculture	50 palm tree garden workers and 50 workers control	Palm tree	Respiratory symptoms including wheezing, sputum, cough and breathless	(43)
	173 farmers	Animal contact	Recurrent wheezing, cough, breathlessness or chronic phlegm	(44)
	433 male and 561 females	Soil	Dyspnea, cough, night cough, night chest discomfort	(45)
Bakers	119 females	Wood tenor	↓ FEV1, FVC and reduced PFT	(46)
	58 bakers	Wood tenor	↑ Respiratory symptoms such as sputum, cough, and wheezing ↓ PFT	(31)
	109 workers	Wood tenor	↑ Dyspnea, cough, night chest discomfort	(47)
	36 workers and 36 workers control	Wood tenor	Change in the pulmonary vascular structure, emphysematous changes in the pulmonary tissue	(48)
	Bakers	Flour factory	Shortness of breath or chest tightness Decreased FEV1, FVC and PFT	(49)
Carpet weavers	66 Iranian carpet weavers	Chemical products	Allergic symptoms and Decrease in FVC and FEV1/FVC	(50)
	–	wool fibers	Reduced respiratory vital capacity	(51)
Factories workers	551 employees	Toluene diisocyanate, polyvinyl chloride, polyethylene or polypropylene	Decreased FEV1, FVC	(52)
	112 cement workers	Cement dust	Decreased FEV1, FVC and reduced PFT	(53)
	170 exposed	Cement producing factory	Significant decreases in vital capacity, FVC, FEF (25–75) and FEV1	(54)
Hairdressers	50 female hairdressers and 50 controls	Whitening powder and hair spray	Respiratory symptoms including wheezing, sputum, cough and breathless	(18)
	200 females and 398 males	Hair color	Increased respiratory symptoms such as: coughing, sputum, wheezing, and sore throat	(55)
	75 beauty salons	Whitening powder	Decreased FEV1, FVC and reduced PFT, increased respiratory symptoms	(56)
Healthcare workers	Healthcare workers such as nurses, physicians, and janitors	Chemical agents such as cleaning and disinfectant substances	Respiratory symptoms	(57)
	37 technicians	Dental laboratories	Decrease in FVC and FEV1/FVC	(58)
Lifeguards	38 male lifeguards and 38 control subjects	Chlorine	Respiratory symptoms and allergic symptoms particularly during the work period	(59)
Miners	67 workers	Stone dust	Respiratory symptoms such as cough, sputum, chest tightness	(24)
Printers	73 printers	–	Enhanced respiratory symptoms	(60)
Veterinary clinic workers	100 veterinary workers	–	Reduced PFT, increased respiratory symptoms	(61)
Workers in slaughterhouses	81 workers	Biological contaminants, such as bacteria and fungi	Decreased FEV1, FVC and reduced PFT, increased respiratory symptoms such as: coughing, sputum, wheezing, and sore throat	(62)
Welders	55 welders	–	Reduced PFT	(63)

FEV1; forced expiratory volume in the first second, FVC; forced vital capacity, RV; residual volume, TLC; total lung capacity; MMEF; maximum mid-expiratory flow, PEF; peak expiratory flow, PFTs; pulmonary function tests; Refs; references.

#### Agriculture

Several chemicals related to agricultural practices are able to induce respiratory disorders. A cross-sectional study was performed on a control group (50 office employees) and a group of 50 subjects working in a palm tree garden from Jahrom, Iran. The results showed higher prevalence of asthma and rhinitis in the garden workers in comparison to the control group. There was no association between asthma, and duration of employment, age, cigarette smoking, water pipe smoking, or other addiction (43).

Another study reported asthma amongst 173 cases including 46 agricultural farmers and 127 sheep breeders in rural areas in northeastern Iran. Higher work-related respiratory and allergic symptoms were observed among sheep breeders compared to agricultural farmers. In addition, the severity of work-related symptoms was worsened by increasing frequency of animal-contact (44).

A cross-sectional study was done in 994 farmers (561 females and 433 males) who were randomly selected from three villages of Isfahan Iran, and evaluated for respiratory symptoms and PFT values including FEV1, FVC, FVC/FEV1 ratio, Peak expiratory flow rate (PEFR) and maximum expiratory flow rate at the middle of the expiration (MEF 25–75). The results of PFT values showed significantly reduction in FEV1 and FEF25–75 among the symptomatic cases. Also, air pollution caused by burning of agricultural residues worsened the pre-existing dyspnea, night chest discomfort, night cough, cough, and exercise-induced cough (45).

#### Bakers

Flour is an organic complex cereal containing dust, which is processed by milling. Normally, flour dust contains different components contributing to dough improvement such as α-amylase, hemicellulose, cellulose, malt enzymes, and additives like baker’s yeast, milk powder, egg powder, and sugar. Wheat flour contains at least 40 allergens representing about 10%–15% of the grain dry weight (26).

The association between flour dust exposure and PFT values was assessed in flour factory workers in Kerman, Iran. More than half of the workers reported sputum after waking up in the morning. Moreover, other workers reported shortness of breath or chest tightness. A considerable negative correlation was also found between FEV1 and FVC and flour dust exposure in the workplace (49).

A prospective study was performed in Tabriz, Iran on bread-baking women with respiratory complaints like COPD (the case group) as well as the women with a definite diagnosis of COPD without a former history of bread baking (the control group). Physical examination, conventional spirometry (FEV1, TLC, and FVC), and pulmonary vascular CT angiography were performed for the patients in both groups. Increased diameter of the bronchial artery, abnormal pulmonary vascular structure, bronchial artery hyper vascularization on radiography, emphysematous alterations in the pulmonary tissue, and reduced FVC and FEV1 were found in the case group in comparison with the control group (48).

The relation between bread baking and incidence of respiratory and allergic symptoms, using a questionnaire among 58 Iranian bakers compared to a control group comprising 58 subjects with other jobs in Mashhad, Iran was examined. In bread bakers, all allergic symptoms like runny nose and sneezing as well as respiratory symptoms such as sputum, cough, and wheezing were higher but their PFT values were significantly lower (64).

A descriptive study was conducted on 109 asthmatic patients referring to Imam Khomeini Hospital in Ardabil, Iran. Using Asthma Control Questionnaire (ACQ). No significant correlation was found between asthma occurrences and baking in wood tenor ovens. However, a significant correlation was found between asthma controls and baking in wood tenor oven. Hence, it is essential to increase community awareness regarding the effects of baking on asthma status (47).

In developing countries, biomass fuels are widely utilized for home heating and cooking, and this may lead to adverse health effects. A relation was reported between the exposure to the byproducts of the biomass fuels combustion, chiefly wood smoke, and respiratory problems. A cross-sectional study was performed on 36 female workers of an industrial bakery who had COPD (mean age of 20 years) and a history of bread baking utilizing biomass fuels in rural places in Tehran, Iran. Low values of PFT including FVC, FEV1, or FEV1/FVC ratio were reported in these women (65).

The reviewed studies reported the incidence of occupational respiratory disorders such as asthma and COPD among bakery workers. It was indicated that the incidence of allergic and respiratory disorders in this group of workers could be reduced by taking safety measures.

#### Carpet weavers

Different lung diseases and reduced respiratory vital capacity are caused by a lack of fresh air in the weaving workshop and the existence of wool fibers together with bio aerosols in the ambient air (51). Wheezing, breathlessness, and cough were reported as the most common symptoms amongst carpet weavers in Mashhad, Iran. Moreover, all respiratory and allergic symptoms were significantly higher while PFT values were significantly lower in comparison to the controls. Furthermore, during working hours, most allergic and respiratory symptoms were significantly more prominent in carpet weavers in comparison to the resting time (50).

#### Carpenters

Respiratory symptoms and PFT values were investigated in 66 carpenters and a matching control group in Mashhad, Iran. Respiratory and allergic symptoms were significantly higher while PFT values were lower in carpenters in comparison to the controls. Moreover, during working hours, the allergic symptoms were worsened. There was a relationship between duration of work and maximal mid-expiratory flow in carpenters. It was also found that these workers were susceptible to occupational asthma (66).

#### Factory workers

Cement dust is a workplace environmental pollution, which could contribute to respiratory insults. The effect of cement dust on PFT values was determined in Ilam, Iran, among 112 cement workers. Gravimetric and x-ray diffraction (XRD) was used to measure total cement dust for determining the solid samples’ SiO_2_ content. None of the workers had any type of damage in the chest or a family or medical history of respiratory illnesses. The results showed a significant reduction in FVC, FEV1, and FEV/FVC ratios in cement workers compared to the control group. Moreover, increased cement dust in the workplace resulted in lower PFT values. Therefore, it is essential to consider cement dust exposure as a main risk factor for chronic respiratory symptoms (53).

In Yazd, Iran, 551 cable-manufacturing employees exposed to cable manufacturing environment (exposed to polyvinyl chloride, toluene isocyanate, polypropylene, or polyethylene) and an unexposed group were studied. A higher incidence of asthma and higher body mass index (BMI) were found in employees exposed to the irritants in the workplace than the control group (52).

In a cross-sectional study in Khash Portland cement producing factory, Iran, PFT values were measured in 170 unexposed and 170 exposed employees. Considerable decreases were observed in FVC, MEF25-75%, and FEV1 in dust-exposed workers in comparison to the unexposed group. According to these findings, a higher prevalence of respiratory symptoms and lung cancer may be caused by occupational exposure to Portland cement dust because of the presence of quartz in cement dusts’ row material. It was also found that cement dust exposure is related to acute and chronic respiratory health effects (54).

#### Hairdressers

Increased prevalence of respiratory symptoms but reduced PFT values were found among the individuals exposed to occupational chemical pollutants in hairdressing salons. Moreover, evidence revealed that in the developing world, inadequate attention is paid to the occupational respiratory risks for hairdressers. It was reported that hair bleaching and using hair sprays are particularly hazardous work-related agents. Increased respiratory symptoms and levels of lung inflammation markers, but reduced PFT values were documented among hairdressers compared to the control populations. Moreover, among female hairdressers (*n* = 200), lower PFT values such as the reduced peak flow rates, were reported in comparison to the males (*n* = 398) randomly selected from different parts of Tehran, Iran (55).

In individuals working in a beauty salon, exposure to air-borne chemicals could potentially lead to respiratory infections and other lung diseases. A study was conducted on the risk of work-based obstructive pulmonary disease in 50 female hairdressers compared to 50 controls from the general population, in Mashhad, Iran, to evaluate respiratory and allergic symptoms, a standard questionnaire was used and PFT values were assessed. The job-related respiratory symptoms were found in almost half of the hairdressers. Cough and chest tightness were the most common symptoms reported among hairdressers and they had significantly higher respiratory symptoms such as wheezing, cough, sputum, and breathlessness in comparison to the control group (18).

A cross-sectional study was performed to assess respiratory and allergic symptoms and PFT values in 75 beauty salons in Malayer and Kangaver, western Iran. The results revealed that respiratory symptoms in hairdressers were mostly caused by exposure to hair spray and whitening powder as allergic chemicals (56). These studies showed higher prevalence of ORD such as asthma among hairdressers, highlighting the need for more strict safety regulations for this job to reduce occupational asthma.

#### Slaughterhouse workers

In a study, respiratory symptoms were assessed among slaughterhouse workers in Shiraz, Iran indicating significant reductions in PFT values including FEV1, FVC, and FEV1/FV amongst these workers. Moreover, the prevalence of cough, breathlessness, productive cough, phlegm, and wheezing among these workers were 17.3%, 27.2%, 37%, 17.3%, and 27.2%, respectively, which were markedly higher compared to the unexposed workers (62).

#### Healthcare workers

There is a high risk of exposure to different chemical agents such as cleaning and disinfectant substances for healthcare workers, which can be potentially hazardous to the respiratory system. Such workers can be protected against exposure to these chemicals in healthcare centers by effectively using respiratory protection equipment. A cross-sectional study was performed on healthcare workers such as nurses, physicians, and janitors in 6 hospitals of Shiraz University of Medical Sciences, Shiraz, Iran. In this work, a questionnaire comprised of 4 components of knowledge, demographic, practice, and perceptions, was used. The results indicated the risk of respiratory hazards as “moderate to high” in most of the participants at their workplaces (57).

#### Lifeguards

Lifeguards are frequently exposed to different irritants during work such as chlorine-inducing irritants, which is able to induce respiratory disorders. A study was performed on a group of 38 male lifeguards and 38 control subjects with similar ages in Mashhad, Iran, and allergic and respiratory symptoms as well as PFT values were assessed. Higher frequencies of work-related respiratory and allergic symptoms in lifeguards mainly during working periods and remarkably lower PFT values were found in lifeguards subjects compared to the control group (59).

#### Miners

Increased prevalence of respiratory disorders was reported in miners. A study was performed on the effect of exposure to stone dust in 67 stone-cutting workers from 35 stone workshops in Kerman province, Iran. The participants had no history of previous chest surgery or respiratory illness. The control group and stone workers had the same age, gender, number of working days per week, average working hours per day, marital status, work experience, education level, smoking, and drug addiction status. Respiratory symptoms such as sputum, cough, and chest tightness were significantly higher in stone-cutting workers compared to the control group. In the workplace, the average annual accumulation of dust was inversely proportional to the PFT values but it was positively related to the respiratory symptoms. Pulmonary infiltration assessed in chest radiology was more prevalent in the stone-cutting worker group. Thus, respiratory diseases and even lung cancer could be caused by exposure to stone dust particles in working environments (24).

#### Printers

A comparison was made in PFT values and respiratory symptoms between 73 printers and 73 subjects with other jobs (considered the control group), in Mashhad, Iran. The results showed significantly increased respiratory symptoms but reduced PFT values in the printer group compared to the control group (60).

#### Veterinary clinic workers

The effect of animal allergen sensitization in animal workers was examined in Shiraz, Iran. In this work, a comparison was made in the PFT values and respiratory symptoms between 100 veterinary workers and 50 controls (non-animal workers). In veterinary workers, PEF and FEV1/FVC were less than 80% of the predicted value, which were lower compared to the control group. It was shown that exposure needs to be minimized and workers should be educated regarding the exposure risks to the sensitizers (61).

#### Welders

A descriptive-analytical study was performed in 55 welders (considered the case group) and 55 non-welders (considered the control group) in Zahedan, Iran. The subjects were selected non-randomly to measure PFT values; all measured spirometry indices were remarkably lower in welders in comparison to the control group. Moreover, there was an inverse association between PFT values and welding history (63). Table 3 shows occupational respiratory disorders (ORD) induced by allergic and irritant agents in the Iran.

### Toxic agents

The toxic agents are most prevalent in factories and coalmines or areas with higher quantities of toxicants including mustard gas, sulfur dioxide, and silica dust, as well as smoke, gases, fumes (28, 29). Considerable airway injury could be induced by environmental such toxic agents. For example, paraquat is a nonselective herbicide, which is sprayed via hand-pressurized backpacks. Oral and nasal irritation and possible absorption may be caused by prolonged direct contact with the mist spray of this agent. Several observational studies have shown the effect of occupational exposure to paraquat on respiratory system (67).

A possible relationship was shown between lung cancer and dust exposure in asbestos-exposed workers of various industry. Some countries have stopped the use of all asbestos types. Nevertheless, extensive use and manufacturing of asbestos products exist in developing countries (68, 69). Table 4 shows ORD induced by toxic agents in Iran.

**Table 4:** Occupational respiratory disorders (ORD) induced by toxic agents in the Iran.

Occupations	Study design	Exposed agents	Effects	Refs
Construction workers	3,000 workers	Asbestos fibers	Lung restrictive functional damages	(70)
Agricultural	126 farmers	PQ	Decreased FEV1, FVC and reduced PFT	(71)
	70 males	PQ	Decreased FEV1, FVC and reduced PFT	(72)
	worker	lead-exposure	Severity of sputum and cough in worker	(73)
Factories workers	–	Crystalline silica	Reduced MMEF and PFT	(74)
	70 workers	Crystalline Silica	Death from lung cancer	(75)
	72 male workers	Silica	Decreased FEV1, FVC and reduced PFT	(76)
	-	Silicosis	Emphysematous changes in the pulmonary tissue, remarkable difference in the PEF parameter	(77)
	-	NO2	Emphysema and Bronchitis	(78)
	Workers	silicosis	Emphysematous changes in the pulmonary tissue	(79)
	110 workers	Cast iron foundry	Decreased FEV1, FVC and reduced PFT	(80)
	263 workers	Ceramic factory	Decreased FEV1, FVC and reduced PFT	(81)
	–	Crystalline silica	Decreased pulmonary function	(82)
		Silica dust	Decreased pulmonary function	(83)
	3,121 workers	Silica-exposed workers	Fibrogenic lung disease	(84)
	–	Silica dust	TB was incremented by the increased intensity of silica exposure	(85)
	127 workers	Dust, metal fumes	Various chronic and acute pulmonary diseases, significant reduction in the lung function parameters	(86)
	263 workers	Tile factory	Decreased PFT values such as FEV1, FVC, and FVC/FEV1	(87)
Medical workers	6 female and 45 males	Methyl methacrylate	Dysfunction and cough, wheezing and shortness of breath	(33)
	37 technicians	Biomedical materials	Coughing, sputum, wheezing, and sore throat	(58)
	42 dental technicians	Methacrylate, nickel and beryllium	Decrease in FVC and FEV1/FVC and shortness of breath	(88)
Miners	Workers	Coal mines	Chronic cough and bronchitis, pneumonitis, and also respiratory infections such as TB are the most common pulmonary disorders	(89)
	556	Coal mines	Reductions in both FEV1 and FVC, increased FEV1/FVC ratio, breathlessness and cough	(19)
	156 workers	kaolin mine, quarry, gold mine	Revealed considerably lower pulmonary function in the exposed groups	(90)
Welders	91 welders	Fume exposure	Reduced MMEF and PFT	(91)
	welders	Particulate Matter	Decreased FEV1, FVC and reduced PFT	(92)

FEV1; forced expiratory volume in the first second, FVC; forced vital capacity, RV; residual volume, TLC; total lung capacity; MMEF; maximum mid-expiratory flow, PEF; peak expiratory flow; PQ; paraquat; TB: tuberculosis; NO2: nitrogen dioxide.

#### Agricultural

A cross-sectional work was performed on respiratory symptoms of 64 male construction workers and 70 male pesticide retailers in Shiraz, Iran. There was an association between the occupational exposure to low doses of pesticides mixture and the incidence of respiratory symptoms and increased blood concentrations of oxidative stress biomarkers (72).

In a cross-sectional investigation, 283 male crop farmers exposed to paraquat and 234 sex-matched healthy individuals were examined. All participants underwent interviews conducted by two trained practitioners. The first questionnaire comprised demographic variables, occupational history, and medical history, while the second questionnaire was the European Community Respiratory Health Survey (ECRHS) questionnaire that aimed to evaluate respiratory symptoms. The findings revealed that a majority of respiratory symptoms were more prevalent in the farmers compared to the reference group, although the difference was statistically significant only for shortness of breath (93). Another cross-sectional study was undertaken on 126 farmers who had been poisoned by paraquat and were referred to Afzalipour Hospital between the years 2006 and 2015 in the Kerman Province of Iran. Various demographic variables, including age and gender, as well as signs and symptoms of poisoning, the estimated amount of paraquat ingested, and the clinical outcome, were derived from the medical records. The findings revealed that the patients with respiratory distress had the highest mortality rate, followed by those with oral ulceration and salivation. Among all the individuals poisoned by paraquat, a dose greater than approximately 2,250 mg was found to predict death with a specificity of 86.2% and a sensitivity of 75.7% (71). In a retrospective study that involved the description, analysis, and assessment of the data, a comprehensive evaluation was performed on 104 medical records of individuals who suffered from paraquat poisoning. These records were obtained from three hospitals located in Shiraz city, Iran. The timeframe for this study spanned from September 2010 to September 2015. The radiographic results from patients who unfortunately did not survive included the presence of acute respiratory distress syndrome (ARDS), pneumothorax, and pneumomediastinum (94). Afzali and colleagues conducted a case–control study to examine the efficacy of immunosuppression on 45 patients who were poisoned with paraquat over a period of 2 years in Hamadan province, Iran. The researchers reached the conclusion that administering pulse therapy with cyclophosphamide and methylprednisolone may potentially be effective in averting respiratory failure and decreasing mortality rates (95). Amiri and colleagues conducted a study that examined 52 instances (farmer) of paraquat poisoning within the province of Lorestan. Their findings revealed a mortality rate of 71.1% attributable to respiratory complications. Furthermore, they deduced that the quantity of paraquat ingested and the duration between the poisoning incident and hospital admission were the principal determinants of mortality (96).

#### Construction workers

Asbestos fibers are found in thermal insulations, chimney pipes and cement sheets and this highlights that workers who work in the demolition of old houses may suffer from the effects of exposing to the asbestos fibers (70). It was revealed that inhalation of asbestos fibers in the air results in developing serious lung diseases including mesothelioma and lung cancer (asbestosis) (97).

##### Factories workers

A cross-sectional study was performed in the southeast of Tehran province, Iran, in 2016. In this study, employees of traditional brick-making workshops (30 workers) and brick-making machine factories (40 workers) were included and their exposure to crystalline silica and total inhalable dust was assessed. The exposure to total respirable dust and crystalline silica was reduced by the mechanization of the industry and reducing the risk of death from lung cancer. However, there was still a higher risk of death from silicosis and lung cancer in both groups of workers (75).

In another cross-sectional study on 72 male workers exposed to silica at a manufacturing plant in Neyshabur, Iran, it was revealed that the average occupational exposure to silica was lower in these concrete workers than international and national organizations’ threshold. However, death and lung cancer risk were still higher than expected values. Moreover, lung indices (FEV1 and FEV1/FVC) were decreased significantly over 4 years employment in these workers (76).

The silicosis risk by crystalline silica exposure, and lung cancer mortality rate were evaluated among male ceramic tile workers in Qazvin, Iran. Higher risk of lung cancer and morality among workers under crystalline silica highlighted an urgent requirement for alterations in rules, assessment, and engineering as well as managerial control approaches in workers of such factories (79).

Non-exposed and dust-exposed groups were compared in another cross-sectional study performed in a cement plant in Kermanshah, Iran. The results revealed that cement dust comprising crystalline silica could lead to respiratory disorders as presented by considerable reductions in the PEF value in the exposed group. Thus, engineering techniques should be optimized to avoid the development of lung diseases like lung cancer, especially in units with higher dust concentrations. Moreover, respiratory masks should be effectively used for minimizing the exposure (77).

In a study performed in 5 ceramic and tile factories between 2009 and 2011 in Yazd, Iran, 100 workers were enrolled from various units of each factory. PFT values were significantly reduced during the 2-year investigation period. The highest reduction was found in FVC in grinding and ball-mill. Reductions in all spirometric parameters were significantly higher in industrial workers in comparison with office workers (98).

A comparison was made on the frequency of respiratory symptoms between 100 controls and 108 lead-exposed workers in Mashhad, Iran. Cough and chest tightness were the most prevalent symptoms in most lead-exposed workers and some of them only reported wheezing. Among lead-exposed workers, most PFT values were significantly lower while lead concentration in their serum and urine was significantly higher than the control group (73).

Nitrogen dioxide (NO_2_) is a toxic gas primarily emitted from burning fossil fuels, vehicle exhausts, and industrial processes. It is a major component of air pollution in urban areas and is known to have detrimental effects on respiratory health. NO_2_ can penetrate into the lungs and cause or worsen COPD, and induce other respiratory diseases such as bronchitis and emphysema. In a study the effect of NO2 on COPD attributed to in people’s lives of Bushehr megacity, in 2011, revealed that COPD is significantly affected by the increased NO_2_ concentration. It was also suggested that the level of NO_2_ can be reduced by electrical, alternative fuels, and solar heating (78).

The respiratory symptoms and PFT values were examined in a case–control study on 40 workers from an automobile factory exposed to different levels of benzene, ethyl-benzene, toluene, and xylene, and 40 unexposed workers (considered the control group) in Tehran, Iran. The workers under air contaminated with benzene, dust, toluene, xylene, ethyl-benzene, and silica, showed significantly lower values of FVC, VC, and PEF compared to the control group indicating induction of respiratory disorders due to exposure to these agents in the environment (99).

A study in Azandarian-Malayer, Iran, in 2006, showed obstructive respiratory diseases based on PFT values (mild to severe) in 16.7% of workers in silica-producing factories. These results are consistent with former studies suggesting that pulmonary function loss can happen with exposure to silica dust, independent of silicosis (100). A prevalence of silicosis of 12.9% mainly in workers over 40 years old and with exposure duration of more than 25 years was reported. This is “long-term exposure” with a moderate quantity of silica dust in the agate industries. Multiple regression analyses showed a relationship between development of silicosis and exposure duration as the only independent predictor which could explain 71.5% of the variance in silicosis development (83).

Tuberculosis is the complication of silicosis that was largely discussed historically over the last centuries. A study on 3,121 workers indicated that silica-exposed workers are at a higher risk of tuberculosis, with or without silicosis. The risk of development of tuberculosis in patients with silicosis is 2.8 to 3.9 times higher than the healthy controls (84). It was reported that occupational silica exposure was most common among tuberculosis patients in a cross-sectional study in Lorestan Province, Iran, in 2012. Moreover, the incidence of tuberculosis was augmented by increased intensity of silica exposure, higher work experience, advanced age, male gender, lower educational level, and cigarette smoking (85).

Various chronic and acute pulmonary diseases are resulted from inhaling hazardous chemical agents like toxic particles and dust, metal fumes, vapors, and gases, as well as other air-borne pollutants, existing in the workplace. A cross-sectional study was performed in workers chosen from the manufacturing unit (*n* = 127, the exposed group) as well as the administrative department (*n* = 30, the unexposed group). All cases were examined using personal air sampling of crystalline silica and pulmonary function tests. A significant reduction in lung function parameters such as the FVC and FEV1 in the silica-exposed workers industries in Saveh, Iran was reported (86).

Assessment of pulmonary parameters of the control and workers in the brick manufacturing industry exposed to dust and respirable crystalline silica revealed significand reduction of FVC%, FEV1/FVC%, FEV1%, and PEF% in workers exposed to dust compared to the control group (101). The PFT values were measured in 263 workers from a tile factory in Semnan, Iran in 2012–2015. It was indicated that most PFT values such as FEV1, FVC, and FVC/FEV1 were significantly decreased in these workers over a three-year period (81). The workers of a tile factory were also studied in Tehran, Iran, and increased respiratory symptoms (sputum, coughing, sore throat, and wheezing) were reported. PFT values were also significantly lower in these workers in comparison to the unexposed workers (87).

The National Institute for Occupational Safety and Health (NIOSH) estimated that over 1.7 million U.S. workers are under crystalline silica. Moreover, more than 250 of them die with silicosis each year (102, 103). Although, no clear profile of exposure to crystalline silica was found among the Iranian workers (104), in a previous study, occupational exposure to crystalline silica clearly showed higher risk of mortality and lung cancer among workers (105).

A cross-shift study in a cast iron foundry in Iran on 90 office workers and 110 workers from the production department, revealed a considerable association between reduction in PFT values in cross-shift workers and dust exposure. Furthermore, PFT values were affected to some degree in workers from the production department compared to the office workers (80).

##### Healthcare workers

Dental technicians are exposed to hazardous substances like nickel, methacrylate, and beryllium (32). Dental technicians (*n* = 42) were examined for the effect of exposure to these agents in Rasht, Iran, and the results revealed increased shortness of breath, prevalence of cough and sputum. Work experience negatively affected the total lung capacity (TLC) and residual volume (RV)/TLC ratio (88). Assessing 51 dental technicians (6 female and 45 male) under methyl methacrylate in Tehran, Iran, revealed that exposure to this chemical, increased wheezing, cough, and shortness of breath in technicians (33).

##### Miners

Coal mining affects the health and mainly the respiratory system of the workers. It is essential to identify potential hazards in coal mines and take suitable measures for minimizing/removing possible exposures (89). In underground mines, exposures are mostly caused by inhalation of particulates, coal dusts, naturally occurring gases, and chemical vapors. The most important airborne hazardous agents are coal dusts as the main inducer of respiratory diseases. Indeed, various respiratory disorders were reported in miners possibly caused by exposure to harmful minerals with a diameter of <10 μm. Chronic lung stroke, pneumoconiosis, lung cancer, benign pleural effusion, inhalation lung injury, reactive dysfunctional airway syndrome, mesothelioma, chronic cough, bronchitis, and asthma were reported in miners. Moreover, the most prevalent pulmonary disorders observed in coal miners are respiratory infections like tuberculosis (89).

A cross-sectional study on 556 coal miners from 3 coal mines of Hashouni, Babnizu, and Hojedk in Tehran, Iran, including 460 workers worked at the coalface for at least 5 years, showed reduced FEV1 and FVC and increased FEV1/FVC ratio in the workers with severe cough and breathlessness. Comparison of non-face and face miners revealed that the disease severity was a function of the dust quantity in the air as well as the cumulative times of inhaling dust by the miners (19). A comparison was made among 156 workers in kaolin mine, quarry, gold mine, and stone cutting workshops (considered the dust-exposed group) and 48 administrative personnel (considered the unexposed group) in Eastern, Iran. The results revealed considerably lower PFT values in the exposed groups (90).

The individuals working in underground and open-pit mines are mainly exposed to silica dust because it is likely to be present in the site soil and released during mining operations courses. In silica dust in mining workers in eastern Iran in 2016–2017, it was indicated that the workers in mining are at higher risk of respiratory diseases, particularly chronic bronchitis and silicosis (106).

##### Welders

A cross-sectional study in an Iranian shipbuilding industrial factory (60 welders and 45 administrative) in 2018, revealed that the metals exist in the welding fusion showed toxicological effects and caused lung cirrhosis induced by iron in these workers. In addition, an inflammatory response may be caused by manganese and chromium, which are known as carcinogens. Moreover, values of FVC, FEV1/FVC, and FEV1 in welders were significantly lower than the administrative staff members (37).

Because of exposure to welding emissions, respiratory complaints/symptoms are common among welders (107). Furthermore, with longer work history, respiratory disorders such as asthma and chest tightness were more prevalent among workers in comparison to the workers with less exposure history. Respiratory complaints particularly chronic bronchitis were more common and the prevalence of respiratory symptoms was higher in welding workers in comparison to the control group. There is no clear information on the exact mechanism of disease development caused by fumes exposure. However, it is thought that an alteration in epithelial cells is caused by inhalation of gases or fumes produced by welding processes or the first line of defense is suppressed (108). Significant post-shift differences were found in FVC and FEV1 between the welders and the control group in a study on 91 welders in some welding tasks. The smoking habit had no significant effects on PFT values among welders in both pre-and post-shift measurements (91).

### Mechanisms of ORD induced by various agents

Lung diseases can be induced by inhalation of irritants, allergic, and toxic agents in the workplace in the exposed workers. More importantly, occupational lung diseases should be clinically recognized as early as possible because they cause serious medical conditions. The host factors involved in the induction of ORD are mostly genes, and polymorphisms and their early identification could be resulted in improvement of ORD (12).

#### Mechanisms of ORD induced by irritant and allergic agents

There is no exact information on the mechanisms behind induction of lung diseases by the irritant and allergic agents. These agents in the worksites can trigger the production of both immunoglobulin E (IgE) and non-IgE and immunological responses in humans (109).

Generally, these agents can induce interleukin (IL)-4 and IL-13 levels, resulting in production of IgE by B cells, following production and secretion of IgE, it binds to basophils and mast cells. Upon activation, soluble allergic mediators are released by these cells like leukotrienes and histamine acting on sensory nerves, smooth muscles, mucous glands, eosinophils, and arteries (110). Vascular permeability, smooth muscle cell contraction, and vasodilation are the commonly increased outcomes of an IgE-mediated reaction that emerge minutes to hours following exposure. Based on the allergen exposure frequency and sites, such reactions may happen in the respiratory system leading to the induction of diseases like allergic rhinitis, asthma, and anaphylaxis (111). Moreover, after sensitization, subsequent exposures lead to eliciting on-IgE-mediated responses causing secretion of pro-inflammatory cytokines such as granulocyte-macrophage colony-stimulating factor (GMCSF), interferon-γ, IL-12, and IL-3, TNF-β activation and recruitment of macrophages and other inflammatory cells (Figure 1) (27).

Allergic gents with HMW acting as complete antigens are innately immunogenic but, it is essential for LMW chemicals to react with heterologous or autologous proteins first to create a hapten-complex before acting as a functioning allergen (27).

LMW allergens have various reactivity, structure, and application. Nevertheless, different common attributes exist related to the immunogenicity such as hyphenation potential, irritancy potential, and capability to access the epithelium. Metal ions like nickel, chromium, and cobalt are among the most common occupationally-relevant allergens (110).

**Figure 3:**
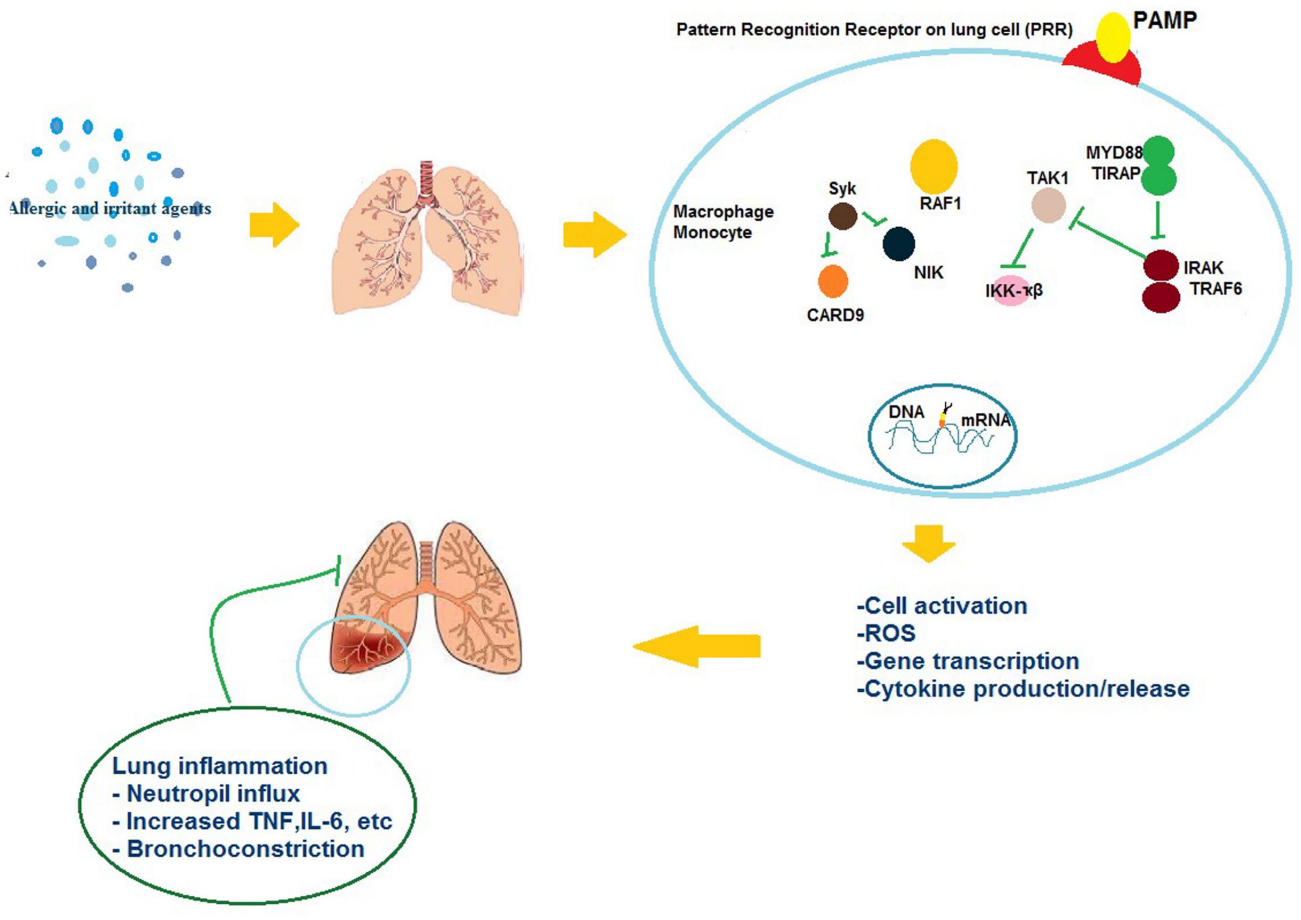
The possible mechanisms of the effects of allergic and irritant agents on respiratory immunologic systems.

Persulfate salts are inorganic salts utilized as oxidizing agents (112). Generally, they are categorized as IgE-mediated sensitizers which also induce a non-IgE-mediated response (113). Although an IgE-mediated mechanism is supported by the animal studies, the role of specific IgE responses in LMW agents-induced asthma is not fully understood. However, further studies are required for evaluating of specific IgG responses to occupational agents (12). Figure 3 shows different mechanisms of ORD induced by irritants and allergic agents.

#### Mechanisms of toxic agent-induced ORD

The indoor pollution sources include mycotoxins, cigarette smoke, and airborne particulates of heavy metals, pesticides, fungicides, asbestos, and silica. COPD may be caused by sustained inflammation of the lung, as occurs against cigarette smoke (29). In cases of inhalation exposure to sublethal quantities of toxicants like mycotoxins, inflammation is resolved usually by eliminating cell injury (114). Formation of chemokines and cytokines by inflammatory and epithelial cells has a key role in the inflammatory procedure. Particularly, TNF-α and IL-1β act as initiator cytokines and induce the production and synthesis of other chemokines, cytokines, and adhesion molecules. Thus, inflammatory cells are attracted and activated to the inflammation site (115).

TNF-α is synthesized initially as a membrane-bound precursor and released proteolytically from cell surfaces (116). Then, soluble TNF-α attaches TNF receptor to activate nuclear factor-kappa B (NF-κB) pathway and mitogen-activated protein kinase (MAPK) cascade. Then, a protein complex is formed by ligand-bound receptor with TNF receptor 1-related death domain protein and TNF receptor-related factor-2 (117, 118). MAPK kinases phosphorylate and activate MAPKs (119). MAPKs phosphorylate and activate transcription factors directly or phosphorylate other kinases, thus, transcription factors are activated resulting in expression of response genes. Other factors involved in several biological processes such as inflammation are also phosphorylated by MAPKs (118).

**Figure 4:**
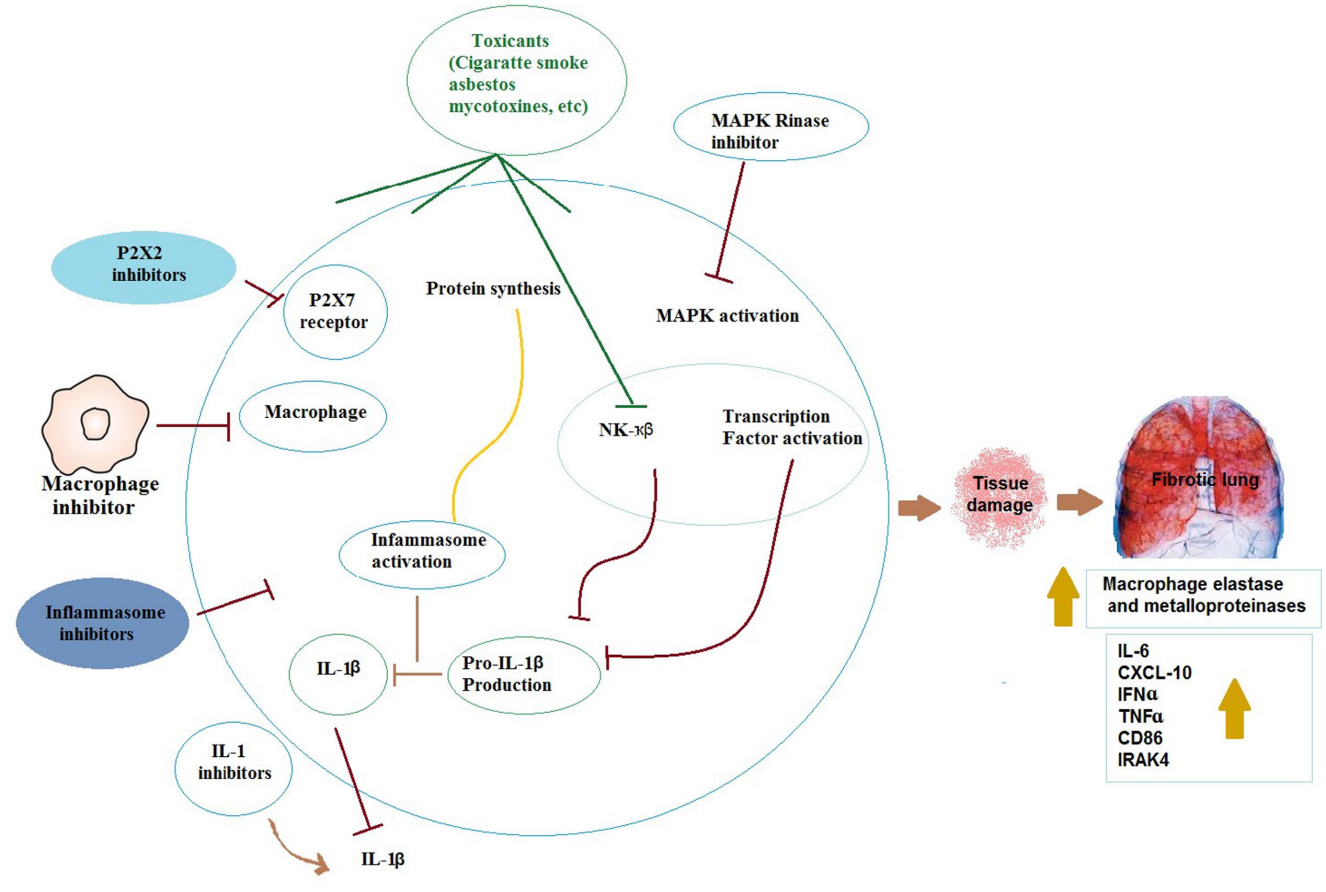
The possible mechanisms of the effects of toxicants on respiratory immunologic systems. IL-1β: Interleukin 1, IL-6: Interleukin 6, TNFɑ: tumor necrosis factor alpha, IFNɑ: Interferon-ɑ, NF-κβ: Nuclear factor kappa β, IRAK4: interleukin-1 receptor-associated kinase 4, CXCL-10: interferon-γ-inducible protein 10.

IL-1β is initially synthesized similar to TNF-α, as pro-IL-1β, an inactive precursor. Then, pro-IL-1β is cleaved within the cell via a protein complex known as the inflammasome comprising apoptosis-based speck-like protein with caspase recruitment domain, caspase-1, and a member of the nucleotide-binding oligomerization domain (NOD)-like receptor family (120). Barious NOD-like receptor members react to different signals. NOD-like receptor protein-3 (NLRP3) as one of these members, is recruited against tissue damage, infection, and metabolic stress (121). By processing pro-IL-1β, mature IL-1β is secreted and bind to IL-1 receptor. A complex is formed by the ligand-bound receptor with myeloid differentiation primary response, TNF receptor-associated factor-6, and IL-1 receptor-associated kinase. Therefore, NF-κB pathway and MAPK cascade are activated (122). Different mechanisms have been shown for activating inflammation process such as generation of reactive oxygen species and potassium efflux (123). Moreover, the importance of P2X7 receptor and autophagy in mediation of the processing of IL-1β by the inflammasome was emphasized (124). These procedures ultimately result in severe lung inflammation causing inflammatory lung disorders. Figure 4 presents different mechanisms involved in toxic agents-induced ORD.

Asbestos causes asbestosis and malignancies by molecular mechanisms that are not fully understood. Asbestos-related lung diseases are a common clinical problem and a major health concern worldwide. Epidemiologic studies have established that exposure to asbestos fibers causes pulmonary fibrosis (asbestosis), pleural abnormalities (effusion and plaques), and malignancies (bronchogenic carcinoma and mesothelioma) (125).

The complete understanding of how asbestos induces epigenetic changes remains elusive. Numerous studies have observed both clastogenic and cytotoxic responses of cells to asbestos fibers (126, 127). The process of phagocytosis of fibers by macrophages and subsequent oxidoreduction reactions on fiber surfaces are known to generate genotoxic reactive oxygen species (ROS), resulting in DNA damage and oxidative stress, ultimately leading to genetic alterations in cells (128, 129). When asbestos interacts with human cells, it attracts and binds cations due to its silicate nature. In the lungs, asbestos fibers retain ions on their surfaces and also release them into the cellular milieu (130). These processes have the potential to generate ROS and free radicals, initiating cellular and DNA damage as well as genotoxicity (131, 132). The high iron content found in certain asbestos fibers, along with the ability of asbestos to adsorb iron *in vivo*, suggests that iron-induced Fenton reactions may contribute to increased ROS levels, inflammation, and carcinogenesis (133). Asbestos is also responsible for inducing an increase in the expression of antioxidant enzymes, including manganese-containing superoxide dismutase (MnSOD, SOD2), catalase, and heme oxygenase, in both malignant mesothelioma cells and the lungs of rodents (125). Types of asbestos have been shown to induce oxidative stress and stimulate the production of local inflammatory mediators, such as cytokines and growth factors, ultimately creating an environment of inflammation and cell proliferation (134). Exposure of cells to asbestos also leads to extensive changes in the expression of genes involved in integrin-mediated signaling, DNA damage repair, and regulation of the cell cycle (135). The chronic inflammation caused by asbestos fibers coming into contact with serosal surfaces is considered a key factor in the development of cancer and is likely mediated through epigenetic alterations (136).

Numerous studies have focused on investigating the role of microRNAs in asbestos-induced epigenetic changes in mesothelioma, a cancer specifically associated with asbestos exposure (137, 138). However, limited research has been conducted on asbestos-related lung cancers, including adenocarcinoma, adenosquamous carcinoma, small cell lung cancer, and large cell lung cancer (139). While these studies demonstrate the potential of asbestos to induce epigenetic alterations, the exact mechanisms by which these factors contribute to cell toxicity and the progression to malignancy remain unclear.

**Figure 5:**
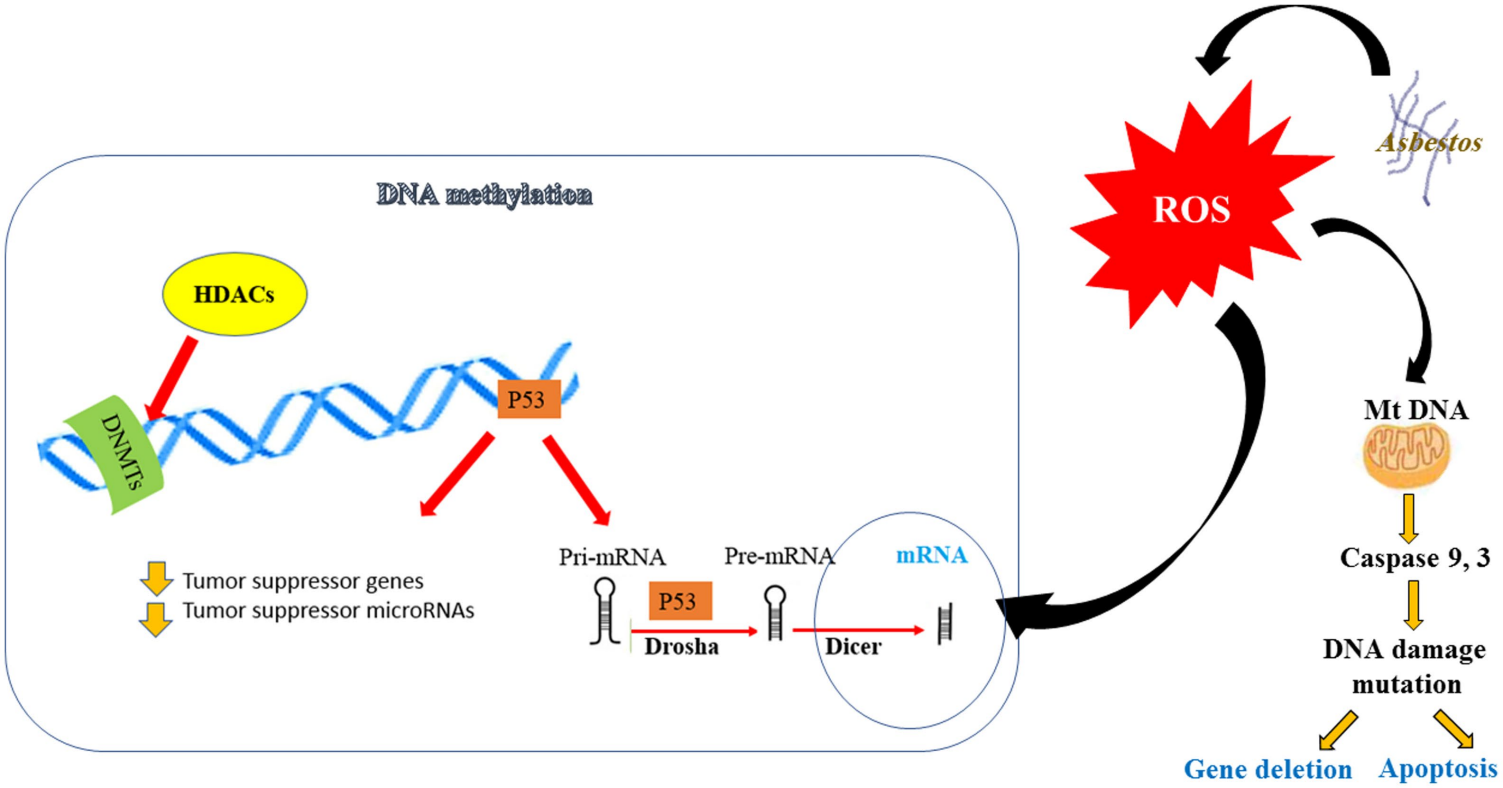
Potential molecular responses of cells to asbestos. When cells are exposed to asbestos, the generation of reactive oxygen species (ROS) will lead to alteration of DNA methylation and microRNA (miRNA) expression/processing, resulting in cell apoptosis or epigenetic alterations that allow cells to progress to diseased states. HDACs: histone deacetylase, DNMTs: DNA methyl transferases, drosha is a class 2 ribonuclease III enzyme that in humans is encoded by the DROSHA gene, Dicer: known as endoribonuclease Dicer or helicase with RNase motif, is an enzyme that in humans is encoded by the DICER1 gene.

Also, studies have demonstrated that epigenetic modifications, particularly DNA methylation, have the potential to effectively identify individuals who are at a heightened risk of developing lung cancer (Figure 5) (140). The connection between promoter DNA hypermethylation and inflammation has been extensively documented in various types of cancer, including lung cancer caused by asbestos exposure (141). It is postulated that the relationship between asbestos exposure and the formation of lung cancer is mediated through this mechanism (136). Genomic investigations have unveiled alterations in DNA copy numbers, modifications in miRNA profiles, and dysregulation of the expression of specific genes in asbestos-related lung cancer (141, 142). Nonetheless, the precise molecular interactions between asbestos fibers and cells, whether direct or indirect, remain largely unknown (143). Figure 5 present potential molecular responses of affected cells to asbestos. When cells are exposed to asbestos, the generation of ROS will lead to alteration of DNA methylation and microRNA (miRNA) expression/processing, resulting in cell apoptosis or epigenetic alterations that allow cells to progress to diseased states.

## Conclusion

In this review, occupational respiratory complications and lung diseases in workers exposed to stimulators and allergens in Iran were discussed. Respiratory complications in mines and factories workers have become more prevalent compared to the general population. Based on the literature, presence of factors that contribute to bronchitis, asthma, lung cancer, tuberculosis in the workplace should be identified as early as possible.

Occupational respiratory disease is considered a preventable disease, and avoidance of exposure to major risk factors can prevent the vast majority of cases specially in developing countries. The present review add to the literature on the prevalence of occupational respiratory disease in Iran and will help policy-makers, specialists, and all stakeholders to strategize and evaluate medical services required for reducing the prevalence of respiratory diseases. Although this review article presented the occupational respiratory disorders in Iran, it will serve as baseline information for future national and regional studies of occupational respiratory disease.

Education of workers about potential occupational pollution should be pay more attention to the safety and health principles of the workplace, factories, and mines. Use of the safety and health systems will help reducing respiratory illnesses in the works and employees in preventing respiratory diseases.

## Author contributions

SB: Methodology, Project administration, Writing – original draft, Writing – review & editing. FA: Investigation, Writing – original draft. HK: Investigation, Methodology, Writing – original draft. MB: Conceptualization, Funding acquisition, Writing – original draft. RR: Writing – review & editing.

## References

[1] Govaerts R Tassignon B Ghillebert J Serrien B de Bock S Ampe T. Prevalence and incidence of work-related musculoskeletal disorders in secondary industries of 21st century Europe: a systematic review and meta-analysis. BMC Musculoskelet Disord. (2021) 22:751–30. doi: 10.1186/s12891-021-04615-9, PMID: 34465326 PMC8408961

[2] Harber P Redlich CA Henneberger PK. Work-related lung diseases. Am J Respir Crit Care Med. (2016) 193:P3–4. doi: 10.1164/rccm.1932P3 26771422 PMC5952360

[3] Goldyn SR Condos R Rom WN. The burden of exposure-related diffuse lung disease. Semin Respir Crit Care Med. (2008) 29:591–602. doi: 10.1055/s-0028-1101269 19221957 PMC3647468

[4] Offermans NS Vermeulen R Burdorf A Goldbohm RA Kauppinen T Kromhout H. Occupational asbestos exposure and risk of pleural mesothelioma, lung cancer, and laryngeal cancer in the prospective Netherlands cohort study. J Occup Environ Med. (2014) 56:6–19. doi: 10.1097/JOM.0000000000000060, PMID: 24351898

[5] Azari MR Yazdian A Zendehdel R Souri H Khodakarim S Peirovi H. Improved method for analysis of airborne asbestos fibers using phase contrast microscopy and FTIR spectrometry. Tanaffos. (2014) 13:38–45. PMID: 25713590 PMC4338051

[6] Sanderson WT Steenland K Deddens JA. Historical respirable quartz exposures of industrial sand workers: 1946–1996. Am J Ind Med. (2000) 38:389–98. doi: 10.1002/1097-0274(200010)38:4<389::AID-AJIM4>3.0.CO;2-J, PMID: 10982979

[7] Driscoll T Nelson DI Steenland K Leigh J Concha-Barrientos M Fingerhut M. The global burden of non-malignant respiratory disease due to occupational airborne exposures. Am J Ind Med. (2005) 48:432–45. doi: 10.1002/ajim.20210, PMID: 16299701

[8] Andersen ZJ De Nazelle A Mendez MA Garcia-Aymerich J Hertel O Tjønneland A. A study of the combined effects of physical activity and air pollution on mortality in elderly urban residents: the Danish diet, Cancer, and health cohort. Environ Health Perspect. (2015) 123:557–63. doi: 10.1289/ehp.1408698, PMID: 25625237 PMC4455593

[9] Clarke K Manrique A Sabo-Attwood T Coker ES. A narrative review of occupational air pollution and respiratory health in farmworkers. Int J Environ Res Public Health. (2021) 18:4097. doi: 10.3390/ijerph18084097, PMID: 33924663 PMC8070429

[10] Jiang X-Q Mei X-D Feng D. Air pollution and chronic airway diseases: what should people know and do? J Thorac Dis. (2016) 8:E31. doi: 10.3978/j.issn.2072-1439.2015.11.50 26904251 PMC4740163

[11] Tarlo SM Broder I. Irritant-induced occupational asthma. Chest. (1989) 96:297–300. doi: 10.1378/chest.96.2.297 2666043

[12] Maestrelli P Boschetto P Fabbri LM Mapp CE. Mechanisms of occupational asthma. J Allergy Clin Immunol. (2009) 123:531–42. doi: 10.1016/j.jaci.2009.01.057 19281901

[13] Dobashi K Akiyama K Usami A Yokozeki H Ikezawa Z Tsurikisawa N. Japanese guideline for occupational allergic diseases 2014. Allergol Int. (2014) 63:421–42. doi: 10.2332/allergolint.14-RAI-0771, PMID: 28942932

[14] Mulugeta Tamene A. Hidat G. Teklehaimanot M. Gebremedhin B. Occupational respiratory health symptoms and associated factor among street sweepers in Addis Ababa, Ethiopia. Occup Med Health Aff. (2017) 5:2. doi: 10.4172/2329-6879.1000262

[15] Sussman GL Beezhold DH. Allergy to latex rubber. Ann Intern Med. (1995) 122:43–6. doi: 10.7326/0003-4819-122-1-199501010-00007 7985895

[16] Hox V Steelant B Fokkens W Nemery B Hellings PW. Occupational upper airway disease: how work affects the nose. Allergy. (2014) 69:282–91. doi: 10.1111/all.12347 24397491

[17] Faghihi-Zarandi AEP Eghbal Sekhavati MR. Occupational exposure to crystalline silica and its pulmonary effects among workers of a cement factory in Saveh, Iran. Indian J Fundam Appl Life Sci. (2015) 5:2822–32.

[18] Hashemi N Boskabady MH Nazari A. Occupational exposures and obstructive lung disease: a case-control study in hairdressers. Respir Care. (2010) 55:895–900. PMID: 20587102

[19] Mahdevari S Shahriar K. A framework for mitigating respiratory diseases in underground coal mining by emphasizing on precautionary measures. Occup Med Health Affairs. (2016) 4:1–6. doi: 10.4172/2329-6879.1000239

[20] Rezapour M Khorrami Z Tabe R Khanjani N. The prevalence of occupational risk factors and occupational diseases in Kerman. Iran Int J Epidemiol Res. (2019) 6:65–9. doi: 10.15171/ijer.2019.12

[21] Sharifi H Masjedi MR Emami H Ghanei M Eslaminejad A Radmand G. Burden of obstructive lung disease study in Tehran: prevalence and risk factors of chronic obstructive pulmonary disease. Lung India. (2015) 32:572–7. doi: 10.4103/0970-2113.168129, PMID: 26664162 PMC4663859

[22] Najmi K Khosravi A Seifi S Emami H Chaibakhsh S Radmand G. Clinicopathologic and survival characteristics of malignant pleural mesothelioma registered in hospital cancer registry. Tanaffos. (2014) 13:6–12. PMID: 25506370 PMC4260067

[23] Emami H Ilbeigi A Khodadad K. An overview of asbestos and malignant pleural mesothelioma: an Iranian perspective. Asian Pac J Cancer Prev. (2017) 18:2619–23. doi: 10.22034/APJCP.2017.18.10.2619, PMID: 29072053 PMC5747379

[24] Ashrafi-Asgarabad A Faculty Member, Faculty of Public Health, Bam Medical University, Bam, Iran, Pulmonologist, Dept of Internal Medicine, Faculty of Medicine, Kerman Medical University, Kerman, Iran, Research Center for Environmental Health Engineering, Faculty of Public Health, Kerman Medical University, Kerman, Iran, Dept of Occupational Health, Faculty of Public Health, Kerman Medical University, Kerman, Iran Samareh-Fekri M khanjani N GhotbiRavandi M. Exposure to particles and respiratory symptoms in stone carvers of Kerman, Iran. J Occup Health Epidemiol. (2013) 2:146–56. doi: 10.18869/acadpub.johe.2.4.146,

[25] GhasemKhani M Kumashiro M Rezaei M Anvari AR Mazloumi A SadeghiPour HR. Prevalence of respiratory symptoms among Workers in Industries of South Tehran, Iran. Indust Health. (2006) 44:218–24. doi: 10.2486/indhealth.44.218, PMID: 16715995

[26] Baatjies R Jeebhay MF. Baker's allergy and asthma-a review of the literature: allergies in the workplace. Curr Allerg Clin Immunol. (2013) 26:232–43.

[27] Anderson SE Long C Dotson GS. Occupational allergy. Europ Med J. (2017) 2:65–71. doi: 10.33590/emj/10311285 PMC6454566 30976662

[28] Berlin A Yodaiken RE Hanman W. Assessment of toxic agents at the workplace: Roles of ambient and biological monitoring. Environ Health Perspect. Springer Science & Business Media (2012) 162:467.

[29] McKay CA. Toxin-induced respiratory distress. Emergency medicine. Clinics. (2014) 32:127–47. doi: 10.1016/j.emc.2013.09.003, PMID: 24275172

[30] Khazraei S Marashi SM Sanaei-Zadeh H. Ventilator settings and outcome of respiratory failure in paraquat-induced pulmonary injury. Sci Rep. (2019) 9:1–5. doi: 10.1038/s41598-019-52939-3 31719587 PMC6851175

[31] Mohammad Hosein Boskabady ET Ahmadi S Ebrahimi K Soudaneh M Mohammadi F Sabourhasanzadeh A. Pulmonary function tests and work-related respiratory and allergic symptoms in Iranian bakers. Iran J Allergy Asthma Immunol. (2009) 8:107–10. 19671940

[32] Kolanz ME. Introduction to beryllium: uses, regulatory history, and disease. Appl Occup Environ Hyg. (2001) 16:559–67. doi: 10.1080/10473220119088, PMID: 11370935

[33] Golbabaei F Mamdouh M Nouri Jelyani K Jamaleddin Shahtaheri S. Exposure to methyl methacrylate and its subjective symptoms among dental technicians, Tehran, Iran. Int J Occup Saf Ergonomics. (2005) 11:283–9. doi: 10.1080/10803548.2005.11076649, PMID: 16219156

[34] Leggat PA Kedjarune U. Toxicity of methyl methacrylate in dentistry. Int Dent J. (2003) 53:126–31. doi: 10.1111/j.1875-595X.2003.tb00736.x 12873108

[35] Baur X. A compendium of causative agents of occupational asthma. J Occup Med Toxicol. (2013) 8:15–8. doi: 10.1186/1745-6673-8-15 23706060 PMC3665602

[36] Nemery B. Metal toxicity and the respiratory tract. Eur Respir J. (1990) 3:202–19. doi: 10.1183/09031936.93.03020202 2178966

[37] Mehrifar Y Zamanian Z Pirami H. Respiratory exposure to toxic gases and metal fumes produced by welding processes and pulmonary function tests. Int J Occup Environ Med. (2019) 10:40–9. doi: 10.15171/ijoem.2019.1540, PMID: 30685776 PMC6522216

[38] Ma Y Deng L Ma P Wu Y Yang X Xiao F. In vivo respiratory toxicology of cooking oil fumes: evidence, mechanisms and prevention. J Hazard Mater. (2021) 402:123455. doi: 10.1016/j.jhazmat.2020.123455, PMID: 32683156

[39] Bakri S Hariri A Ismail M. Metal fumes toxicity and its association with lung health problems among welders in automotive industry. J Phys. (2019) 1150:012001. doi: 10.1088/1742-6596/1150/1/012001

[40] Kogevinas M Antó JM Sunyer J Tobias A Kromhout H Burney P. Occupational asthma in Europe and other industrialised areas: a population-based study. Lancet. (1999) 353:1750–4. doi: 10.1016/S0140-6736(98)07397-8 10347988

[41] Arif AA Delclos GL Whitehead LW Tortolero SR Lee ES. Occupational exposures associated with work-related asthma and work-related wheezing among US workers. Am J Ind Med. (2003) 44:368–76. doi: 10.1002/ajim.10291, PMID: 14502764

[42] Baur X Bakehe P Vellguth H. Bronchial asthma and COPD due to irritants in the workplace-an evidence-based approach. J Occup Med Toxicol. (2012) 7:19–31. doi: 10.1186/1745-6673-7-19 23013890 PMC3508803

[43] Farahmand MA Khanjani N Mianroodi AAA Asgarabad AT. Relationship between Asthma and Allergic Rhinitis with Working in Palm Tree Gardens in Jahrom City in 2016. Iran J Aller Asthma Immunol. (2018) 17:176.

[44] Hashemi N Mirsadraee M Shakeri M Varasteh A. Prevalence of work-related respiratory symptoms in Iranian farmers. Can Respir J. (2006) 13:198–202. doi: 10.1155/2006/967895, PMID: 16779464 PMC2683279

[45] Golshan M Faghihi M Roushan-Zamir T Marandi MM Esteki B Dadvand P. Early effects of burning rice farm residues on respiratory symptoms of villagers in suburbs of Isfahan. Iran Int J Environ Health Res. (2002) 12:125–31. doi: 10.1080/09603120220129283, PMID: 12396529

[46] Ahmadi S Moradians V Javad Moosavi SA Kouranifar S Momeni MK. The effect of inhaled salbutamol on residual capacity in patients with chronic obstructive pulmonary disease. J Iran Med Council. (2019) 2:92–7.

[47] Dashti S Zare L Shahmari M Dashti F Dashti A. Association between tobacco smoke exposure (environmental and direct) and incidence and control of bronchial asthma. Open J Nurs. (2018) 8:150–6. doi: 10.4236/ojn.2018.82013

[48] Tarzamni MK Ghaffari MM Javadrashid MR Arablou F Mostafaei A. Investigating types and frequency of vascular findings in pulmonary CT angiography of baker females with pulmonary complaints in comparison with chronic obstructive pulmonary disease. Am J Life Sci Res. (2017) 5:51–61. doi: 10.21859/ajlsr-05023

[49] Bagheri Hosseinabadi M Krozhdeh J Khanjani N Zamani A Ranjbar M Mohammadian M. Relationship between lung function and flour dust in flour factory workers. J Commun Health Res. (2013) 2:138–46.

[50] Boskabady MH Karimiani EG Vostacolaei HA. Respiratory symptoms and pulmonary function changes among carpet weavers in Iran. Int J Occup Environ Health. (2007) 13:369–75. doi: 10.1179/oeh.2007.13.4.369, PMID: 18085050

[51] Rafeemanesh E Khooei A Niroumand S Shirzadeh T. A study on musculoskeletal complaints and working postures in pathology specialists in Iran. BMC Musculoskelet Disord. (2021) 22:1012–8. doi: 10.1186/s12891-021-04870-w, PMID: 34861852 PMC8642988

[52] Attarchi M Dehghan F Yazdanparast T Mohammadi S Golchin M Sadeghi Z. Occupational asthma in a cable manufacturing company. Iran Red Crescent Med J. (2014) 16:e9105. doi: 10.5812/ircmj.9105, PMID: 25558389 PMC4270639

[53] Poornajaf A Kakooei H Hosseini M Ferasati F Kakaei H. The effect of cement dust on the lung function in a cement factory. Iran Int J Occup Hygiene. (2010) 2:74–8.

[54] Mirzaee R Kebriaei A Hashemi S Sadeghi M Shahrakipour M. Effects of exposure to Portland cement dust on lung function in Portland cement factory workers in Khash, Iran. J Environ Health Sci Eng. (2008) 5:201–6.

[55] Mahmoudi F. Occupational health problems of hairdressers of Tehran. Acta Med Iran. (1996) 34:14–6.

[56] Almasi A Dargahi A Mohammadi M Amirian F Shokri A Tabandeh L. Comparative study of awareness, attitude, and performance of hairdressers in west regions of Iran in terms of personal hygiene, decontamination of tools and devices, and general status of building. J Chem Pharm Sci. (2016) 9:3056–62.

[57] Honarbakhsh M Jahangiri M Ghaem H. Knowledge, perceptions and practices of healthcare workers regarding the use of respiratory protection equipment at Iran hospitals. J Infect Prev. (2018) 19:29–36. doi: 10.1177/1757177417724880, PMID: 29317912 PMC5753949

[58] Hatami M Mehrpaevar A. Spirometric evaluation of pulmonary function in Yazd’s dental laboratory workers in 1398. Occup Med. (2020). doi: 10.18502/tkj.v12i3.4986

[59] Boskabady M Esmaeilizadeh M Boskabady M. The effect of exposure to chlorine on pulmonary function tests and respiratory and allergic symptoms in Iranian lifeguards. Toxicol Ind Health. (2014) 30:218–24. doi: 10.1177/0748233712454465 22851523

[60] Boskabady MH Rezaiyan MK Navabi I Shafiei S Shafiei S. Work-related respiratory symptoms and pulmonary function tests in Iranian printers. Saudi Med J. (2009) 30:1170–5. PMID: 19750262

[61] Moghtaderi M Farjadian S Abbaszadeh Hasiri M. Animal allergen sensitization in veterinarians and laboratory animal workers. Occup Med. (2014) 64:516–20. doi: 10.1093/occmed/kqu097 25104279

[62] Kasaeinasab A Jahangiri M Karimi A Tabatabaei HR Safari S. Respiratory disorders among workers in slaughterhouses. Saf Health Work. (2017) 8:84–8. doi: 10.1016/j.shaw.2016.04.002, PMID: 28344845 PMC5355534

[63] Komeili G. Mirzaei R. Nabizadeh S. S. Comparative study of lung functional tests in Zahedan welders. Health Scope. (2013) 2:145–8.

[64] Boskabadi M. Taheri E. Ahmadi S. Ebrahimi K. Soudaneh M. Mohammadi F.. Pulmonary function tests and work-related respiratory and allergic symptoms in Iranian bakers. (2009). 19671940

[65] Yazdanpanah L Shidfar F Moosavi J Heidarnazhad H Haghani H. Assessment of nutritional status in chronic obstructive pulmonary disease patients. Iran J Public Health. (2009) 38:39–45.

[66] Boskabady MH Rezaiyan MK Navabi I Shafiei S Arab SS. Work-related respiratory symptoms and pulmonary function tests in northeast iranian (the city of Mashhad) carpenters. Clinics. (2010) 65:1003–7. doi: 10.1590/S1807-59322010001000013, PMID: 21120301 PMC2972615

[67] Wegman D Peters J Boundy MG Smith T. Evaluation of respiratory effects in miners and millers exposed to talc free of asbestos and silica. Occup Environ Med. (1982) 39:233–8. doi: 10.1136/oem.39.3.233, PMID: 7093149 PMC1009016

[68] Kakooei H Sameti M Kakooei AA. Asbestos exposure during routine brake lining manufacture. Ind Health. (2007) 45:787–92. doi: 10.2486/indhealth.45.787, PMID: 18212474

[69] Marioryad H Kakooei H Shahtaheri SJ Yunesian M Azam K. Assessment of airborne asbestos exposure at an asbestos cement sheet and pipe factory in Iran. Regul Toxicol Pharmacol. (2011) 60:200–5. doi: 10.1016/j.yrtph.2011.03.005, PMID: 21420461

[70] Kakooei H Normohammadi M. Asbestos exposure among construction workers during demolition of old houses in Tehran, Iran. Indus Health. (2013) 52:71–7. doi: 10.2486/indhealth.2012-0118 PMC4202766 24292876

[71] Oghabian Z Williams J Mohajeri M Nakhaee S Shojaeepour S Amirabadizadeh A. Clinical features, treatment, prognosis, and mortality in paraquat poisonings: a hospital-based study in Iran. J Res Pharm Pract. (2019) 8:129. doi: 10.4103/jrpp.JRPP_18_71 31728343 PMC6830018

[72] Jalilian H Neghab M Tatar M Taheri S. Respiratory and dermal symptoms and raised serum concentrations of biomarkers of oxidative stress among pesticide retailers. Int J Occup Environ Med. (2018) 9:194–204. doi: 10.15171/ijoem.2018.1417, PMID: 30325360 PMC6466989

[73] Khazdair MR Boskabady MH Afshari R Dadpour B Behforouz A Javidi M. Respiratory symptoms and pulmonary function testes in lead exposed workers. Iran Red Crescent Med J. (2012) 14:738–43. doi: 10.5812/ircmj.4134 PMC3560546 23396762

[74] Gharavandi S Nasori M Ghobadi M Besharatipur M Jabari M Omidianidost A. Exposure to respirable dust and crystalline silica in a cement plant. Arch Occup Health. (2019) 3:366–70. doi: 10.18502/aoh.v3i3.1279

[75] Rezazahehazari M Sahatfardi F Zarei F Ebrahimi Hariri A Salehpour S Soori H. Risk assessment of mortality from silicosis and lung cancer in workers of machine factories and traditional brick production workshops with crystalline silica exposure. Occup Med. (2020) 26–34. doi: 10.18502/tkj.v12i3.4984

[76] Moghadam SR Khanjani N Mohamadyan M Emkani M Yari S Tizabi MNL. Changes in spirometry indices and lung cancer mortality risk estimation in concrete workers exposed io crystalline silica. Asian Pacific J Cancer Prev. (2020) 21:2811–7. doi: 10.31557/APJCP.2020.21.9.2811, PMID: 32986385 PMC7779439

[77] Omidianidost A Gharavandi S Azari MR Hashemian AH Ghasemkhani M Rajati F. Occupational exposure to respirable dust, crystalline silica and its pulmonary effects among workers of a cement factory in Kermanshah, Iran. Tanaffos. (2019) 18:157–62. PMID: 32440304 PMC7230124

[78] Saki H Goudarzi G Jalali S Barzegar G Farhadi M Parseh I. Study of relationship between nitrogen dioxide and chronic obstructive pulmonary disease in Bushehr. Iran Clin Epidemiol Glob Health. (2020) 8:446–9. doi: 10.1016/j.cegh.2019.10.006

[79] Nourmohammadi M Asadi AF Jarrahi AM Yari S. Risk of mortality caused by silicosis and lung Cancer: a study on ceramic tile factory workers. Asian Pacific J Environ Cancer. (2018) 1:55–8. doi: 10.31557/apjec.2018.1.1.55-58

[80] Saraei M Masoudi H Aminian O Izadi N. Respiratory health and cross-shift changes of foundry Workers in Iran. Tanaffos. (2018) 17:285–90. 31143220 PMC6534795

[81] Pahlevan D Mirmohammadkhani M Farzaneh A Mansori K Khazaei S Malek F. Assessment of the changes of spirometric parameters and factors associated in tiles and ceramic factory workers of Semnan city (Iran). Egypt J Chest Dis Tuberc. (2020) 69:747. doi: 10.4103/ejcdt.ejcdt_16_20

[82] Aghilinejad M Jamaati H Farshad A Mostafaie M Shidfar F Atari G. Investigation of prevalence rate of silicosis in silica powderproduction workers in Azandarian-Malayer in 2001-2002. Iran Occup Health. (2006) 3:76–80.

[83] Mohebbi I Khaled Rezaei M. EpidemiologicalAssessment of silicosis in stone cutting workers. Iran Occup Health. (2006) 3:10.

[84] Farazi A Jabbariasl M. Silico-tuberculosis and associated risk factors in central province of Iran. Pan Afr Med J. (2015) 20:333. doi: 10.11604/pamj.2015.20.333.4993, PMID: 26175823 PMC4491449

[85] Yarahmadi A Zahmatkesh MM Ghaffari M Mohammadi S Labbafinejad Y Seyedmehdi SM. Correlation between silica exposure and risk of tuberculosis in Lorestan Province of Iran. Tanaffos. (2013) 12:34–40. PMID: 25191460 PMC4153244

[86] Mohammadi H Dehghan SF Golbabaei F Roshani S Pakzad R Foroughi P. Pulmonary functions and health-related quality of life among silica-exposed workers. Tanaffos. (2017) 16:60–7. PMID: 28638426 PMC5473384

[87] Dehghan F. Mohammadi S. Sadeghi Z. ATARCHI M. Respiratory complaints and spirometric parameters in tile and ceramic factory workers. Tanaffos. (2009) 8:19–25.

[88] Alavi A Shakiba M Nejad AT Massahnia S Shiari A. Respiratory findings in dental laboratory technicians in Rasht (north of Iran). Tanaffos. (2011) 10:44–9. PMID: 25191362 PMC4153148

[89] Mahdevari S Shahriar K Esfahanipour A. Human health and safety risks management in underground coal mines using fuzzy TOPSIS. Sci Total Environ. (2014) 488-489:85–99. doi: 10.1016/j.scitotenv.2014.04.076 24815558

[90] Gholami A Sajedifar J Tazeroudi A Attar M. Lung function and respiratory symptoms among mine workers in the eastern part of Iran. Russian Open Med J. (2018) 7:1–4. doi: 10.15275/rusomj.2018.0306

[91] Golbabaei F Khadem M Ghahri A Babai M Hosseini M SeyedSomea M. Pulmonary functions of welders in gas transmission pipelines in Iran. Int J Occup Saf Ergon. (2013) 19:647–55. doi: 10.1080/10803548.2013.11077008, PMID: 24321643

[92] Khaefi M Geravandi S Hassani G Yari AR Soltani F Dobaradaran S. Association of particulate matter impact on prevalence of chronic obstructive pulmonary disease in Ahvaz, Southwest Iran during 2009-2013. Aerosol Air Qual Res. (2017) 17:230–7. doi: 10.4209/aaqr.2015.11.0628

[93] Mokarami H Choobineh S Rahimian F Soleimani E. Respiratory symptoms among crop farmers and comparison with a general population sample: a cross-sectional study. Toxicol Environ Heal Sci. (2022) 14:187–92. doi: 10.1007/s13530-022-00128-7

[94] Kavousi-Gharbi S Jalli R Rasekhi-Kazerouni A Habibagahi Z Marashi SM. Discernment scheme for paraquat poisoning: a five-year experience in shiraz, Iran. World J Exp Med. (2017) 7:31–9. doi: 10.5493/wjem.v7.i1.31, PMID: 28261553 PMC5316902

[95] Afzali S. Gholyaf M. The effectiveness of combined treatment with methylprednisolone and cyclophosphamide in oral paraquat poisoning. Arch Iran Med. (2008) 11:387–91. 18588370

[96] Amiri AH Delfan B Jaferian S. Paraquat poisoning cases treated at Shohada Ashayer hospital of Khorramabad in 2001-2006. Res J Biol Sci. (2008) 3:525–9.

[97] Taghizadeh F Jafari AJ Gholami M Kermani M Arfaeinia H Mohammadi S. Monitoring of airborne asbestos fibers in an urban ambient air of Shahryar City, Iran: levels, spatial distribution, seasonal variations, and health risk assessment. Environ Sci Pollut Res. (2019) 26:6450–9. doi: 10.1007/s11356-018-4029-0, PMID: 30623323

[98] Mehrparvar A Mirmohammadi S Mostaghaci M Davari M Hashemi S. A 2-year follow-up of spirometric parameters in workers of a tile and ceramic industry, Yazd, southeastern Iran. Int J Occup Environ Med. (2013) 4:73–9. 23567532

[99] Harati B Shahtaheri SJ Karimi A Azam K Ahmadi A Rad MA. Evaluation of respiratory symptoms among workers in an automobile manufacturing factory, Iran. Iran J Public Health. (2018) 47:237–45. PMID: 29445634 PMC5810387

[100] Aghilinezhad M. Jamaati H. Farshad A. Mostafaei M. Shidfar F. Atari G. Investigation of prevalence rate of silicosis in silica powder production workers in Azandarian-Malayer in 2001–2002. Iran Occup Health. (2006) 3:76–80.

[101] Saranjam B. Validation of an adapted NIOSH method for analysis of airborne crystalline silica. Shahid Beheshti University of Medical Sciences, School of Public Health. NIOSH Manual of Analytical Methods (NMAM). (2014).

[102] Baron P. A. Rice F. L. Key-Schwartz R. Bartley D. Schlecht P. Health effects of occupational exposure to respirable crystalline silica. National Institute for Occupational Safety and Health. (2002).

[103] Golbabaei F Faghihi A Ebrahimnezhad P Banshi M Mohseni H Shokri A. Assessment of occupational exposure to the respirable fraction of cement dust and crystalline silica. J Health Saf Work. (2012) 2:17–28.

[104] ZareiFAzariMRSalehpourSKhodakarimSOmidiLTavakolE. Respiratory effects of simultaneous exposure to respirable crystalline silica dust, formaldehyde, and triethylamine of a group of foundry workers. J Res Health Sci. (2017) 17:371.28413169

[105] Mohammadyan M Rokni M Yosefinejad R. Occupational exposure to respirable crystalline silica in the Iranian Mazandaran province industry workers. Arh Hig Rada Toksikol. (2013) 64:139–43. doi: 10.2478/10004-1254-64-2013-2284 23585166

[106] Golbabaei F Gholami A Teimori-Boghsani G Yaseri M Kianmehr M. Evaluation of occupational exposure to silica dust in mining workers in eastern Iran. Open Environ Res J. (2019) 12:1–6. doi: 10.2174/1874213001912010001

[107] Loukzadeh Z Sharifian SA Aminian O Shojaoddiny-Ardekani A. Pulmonary effects of spot welding in automobile assembly. Occup Med. (2009) 59:267–9. doi: 10.1093/occmed/kqp033, PMID: 19286991

[108] Rahmani AH Alhorabi A Josef J Babiker A. Study of work related respiratory symptoms among welding workers. Asian J Pharm Clin Res. (2018) 11:97–9. doi: 10.22159/ajpcr.2018.v11i2.22767

[109] Brashier B Wankhede A. Occupational asthma In: Vedanthan PK Nelson HS Agashe SN Mahesh PA Katial R, editors. Textbook of Allergy for the Clinician: CRC Press (2021). 241–54.

[110] Scheman A Severson D. American contact dermatitis society contact allergy management program: an epidemiologic tool to quantify ingredient usage. Dermatitis. (2016) 27:11–3. doi: 10.1097/DER.0000000000000152, PMID: 26756510

[111] Galli SJ Tsai M Piliponsky AM. The development of allergic inflammation. Nature. (2008) 454:445–54. doi: 10.1038/nature07204, PMID: 18650915 PMC3573758

[112] Pang S Fiume M. Final report on the safety assessment of ammonium, potassium, and sodium persulfate. Int J Toxicol. (2001) 20:7–21. doi: 10.1080/10915810152630710, PMID: 11766134

[113] De Vooght V Cruz M-J Haenen S Wijnhoven K Muñoz X Hoet PH. Ammonium persulfate can initiate an asthmatic response in mice. Thorax. (2010) 65:252–7. doi: 10.1136/thx.2009.121293, PMID: 20335296

[114] Brusselle G Bracke K. Targeting immune pathways for therapy in asthma and chronic obstructive pulmonary disease. Ann Am Thorac Soc. (2014) 11:S322–8. doi: 10.1513/AnnalsATS.201403-118AW 25525740

[115] Kota S Sabbah A Harnack R Xiang Y Meng X Bose S. Role of human β-defensin-2 during tumor necrosis factor-α/NF-κB-mediated innate antiviral response against human respiratory syncytial virus. J Biol Chem. (2008) 283:22417–29. doi: 10.1074/jbc.M710415200, PMID: 18567888 PMC2504899

[116] Horiuchi K Kimura T Miyamoto T Takaishi H Okada Y Toyama Y. Cutting edge: TNF-α-converting enzyme (TACE/ADAM17) inactivation in mouse myeloid cells prevents lethality from endotoxin shock. J Immunol. (2007) 179:2686–9. doi: 10.4049/jimmunol.179.5.2686, PMID: 17709479

[117] Darnay BG Aggarwal BB. Early events in TNF signaling: a story of associations and dissociations. J Leukoc Biol. (1997) 61:559–66. doi: 10.1002/jlb.61.5.559, PMID: 9129204

[118] Liu ZG. Molecular mechanism of TNF signaling and beyond. Cell Res. (2005) 15:24–7. doi: 10.1038/sj.cr.7290259 15686622

[119] Chang L Karin M. Mammalian MAP kinase signalling cascades. Nature. (2001) 410:37–40. doi: 10.1038/35065000 11242034

[120] Sedger LM McDermott MF. TNF and TNF-receptors: from mediators of cell death and inflammation to therapeutic giants–past, present and future. Cytokine Growth Factor Rev. (2014) 25:453–72. doi: 10.1016/j.cytogfr.2014.07.016, PMID: 25169849

[121] Latz E Xiao TS Stutz A. Activation and regulation of the inflammasomes. Nat Rev Immunol. (2013) 13:397–411. doi: 10.1038/nri3452, PMID: 23702978 PMC3807999

[122] Liew FY Xu D Brint EK O'Neill LA. Negative regulation of toll-like receptor-mediated immune responses. Nat Rev Immunol. (2005) 5:446–58. doi: 10.1038/nri1630 15928677

[123] Bauernfeind F Bartok E Rieger A Franchi L Núñez G Hornung V. Cutting edge: reactive oxygen species inhibitors block priming, but not activation, of the NLRP3 inflammasome. J Immunol. (2011) 187:613–7. doi: 10.4049/jimmunol.1100613, PMID: 21677136 PMC3131480

[124] Labasi J Perregaux DG Stam E Petrushova N Griffiths RJ Gabel CA. Altered cytokine production in mice lacking P2X7Receptors. J Biol Chem. (2001) 276:125–32. doi: 10.1074/jbc.M006781200 11016935

[125] Heintz NH Janssen-Heininger YM Mossman BT. Asbestos, lung cancers, and mesotheliomas: from molecular approaches to targeting tumor survival pathways. Am J Respir Cell Mol Biol. (2010) 42:133–9. doi: 10.1165/rcmb.2009-0206TR, PMID: 20068227 PMC2822975

[126] Msiska Z Pacurari M Mishra A Leonard SS Castranova V Vallyathan V. DNA double-strand breaks by asbestos, silica, and titanium dioxide: possible biomarker of carcinogenic potential? Am J Respir Cell Mol Biol. (2010) 43:210–9. doi: 10.1165/rcmb.2009-0062OC 19783790

[127] Vaslet CA Messier NJ Kane AB. Accelerated progression of asbestos-induced mesotheliomas in heterozygous p53+/− mice. Toxicol Sci. (2002) 68:331–8. doi: 10.1093/toxsci/68.2.331, PMID: 12151629

[128] Pietruska JR Johnston T Zhitkovich A Kane AB. XRCC1 deficiency sensitizes human lung epithelial cells to genotoxicity by crocidolite asbestos and Libby amphibole. Environ Health Perspect. (2010) 118:1707–13. doi: 10.1289/ehp.1002312, PMID: 20705543 PMC3205592

[129] Cheng YY Rath EM Linton A Yuen ML Takahashi K Lee K. The current understanding of asbestos-induced epigenetic changes associated with lung cancer. Lung Cancer (Auckl). (2020) 11:1–11. doi: 10.2147/LCTT.S186843 32021524 PMC6955579

[130] Baldys A Aust AE. Role of iron in inactivation of epidermal growth factor receptor after asbestos treatment of human lung and pleural target cells. Am J Respir Cell Mol Biol. (2005) 32:436–42. doi: 10.1165/rcmb.2004-0133OC, PMID: 15626777

[131] Aljandali A Pollack H Yeldandi A Yuyu L Weitzman SA Kamp DW. Asbestos causes apoptosis in alveolar epithelial cells: role of iron-induced free radicals. J Lab Clin Med. (2001) 137:330–9. doi: 10.1067/mlc.2001.114826, PMID: 11329530

[132] Upadhyay D Kamp DW. Asbestos-induced pulmonary toxicity: role of DNA damage and apoptosis. Exp Biol Med. (2003) 228:650–9. doi: 10.1177/153537020322800602, PMID: 12773695

[133] Liu G Cheresh P Kamp DW. Molecular basis of asbestos-induced lung disease. Annu Rev Pathol. (2013) 8:161–87. doi: 10.1146/annurev-pathol-020712-163942, PMID: 23347351 PMC3900296

[134] Nymark P Lindholm PM Korpela MV Lahti L Ruosaari S Kaski S. Gene expression profiles in asbestos-exposed epithelial and mesothelial lung cell lines. BMC Genomics. (2007) 8:1–14. doi: 10.1186/1471-2164-8-62 17331233 PMC1821332

[135] Acencio MMP Soares B Marchi E Silva CSR Teixeira LR Broaddus V. Inflammatory cytokines contribute to asbestos-induced injury of mesothelial cells. Lung. (2015) 193:831–7. doi: 10.1007/s00408-015-9744-4, PMID: 26059286

[136] Perwez Hussain S Harris CC. Inflammation and cancer: an ancient link with novel potentials. Int J Cancer. (2007) 121:2373–80. doi: 10.1002/ijc.23173, PMID: 17893866

[137] Kirschner MB Cheng YY Armstrong NJ Lin RC Kao SC Linton A. MiR-score: a novel 6-microRNA signature that predicts survival outcomes in patients with malignant pleural mesothelioma. Mol Oncol. (2015) 9:715–26. doi: 10.1016/j.molonc.2014.11.007 25497279 PMC5528709

[138] Johnson TG Schelch K Cheng YY Williams M Sarun KH Kirschner MB. Dysregulated expression of the MicroRNA miR-137 and its target YBX1 contribute to the invasive characteristics of malignant pleural mesothelioma. J Thorac Oncol. (2018) 13:258–72. doi: 10.1016/j.jtho.2017.10.016 29113949

[139] Nymark P Guled M Borze I Faisal A Lahti L Salmenkivi K. Integrative analysis of microRNA, mRNA and aCGH data reveals asbestos-and histology-related changes in lung cancer. Genes Chromosom Cancer. (2011) 50:585–97. doi: 10.1002/gcc.20880 21563230

[140] Wang H Gillis A Zhao C Lee E Wu J Zhang F. Crocidolite asbestos-induced signal pathway dysregulation in mesothelial cells. Mutat Res. (2011) 723:171–6. doi: 10.1016/j.mrgentox.2011.04.008 21570478

[141] Kettunen E Hernandez-Vargas H Cros MP Durand G Le Calvez-Kelm F Stuopelyte K. Asbestos-associated genome-wide DNA methylation changes in lung cancer. Int J Cancer. (2017) 141:2014–29. doi: 10.1002/ijc.30897, PMID: 28722770

[142] Mossman BT Lippmann M Hesterberg TW Kelsey KT Barchowsky A Bonner JC. Pulmonary endpoints (lung carcinomas and asbestosis) following inhalation exposure to asbestos. J Toxicol Environ Health. (2011) 14:76–121. doi: 10.1080/10937404.2011.556047, PMID: 21534086 PMC3118517

[143] Nymark P Aavikko M Mäkilä J Ruosaari S Hienonen-Kempas T Wikman H. Accumulation of genomic alterations in 2p16, 9q33. 1 and 19p13 in lung tumours of asbestos-exposed patients. Mol Oncol. (2013) 7:29–40. doi: 10.1016/j.molonc.2012.07.006, PMID: 22901466 PMC5528398

